# Direction-Specific Iterative Tuning of Motor Commands With Local Generalization During Randomized Reaching Practice Across Movement Directions

**DOI:** 10.3389/fnbot.2021.651214

**Published:** 2021-10-29

**Authors:** Pritesh N. Parmar, James L. Patton

**Affiliations:** ^1^Richard and Loan Hill Department of Bioengineering, University of Illinois at Chicago, Chicago, IL, United States; ^2^Shirley Ryan AbilityLab (formerly the Rehabilitation Institute of Chicago), Chicago, IL, United States

**Keywords:** novel motor skill learning, internal model, error-feedback, visuomotor adaptation, error-augmentation, generalization, randomized training

## Abstract

During motor learning, people often practice reaching in variety of movement directions in a randomized sequence. Such training has been shown to enhance retention and transfer capability of the acquired skill compared to the blocked repetition of the same movement direction. The learning system must accommodate such randomized order either by having a memory for each movement direction, or by being able to generalize what was learned in one movement direction to the controls of nearby directions. While our preliminary study used a comprehensive dataset from visuomotor learning experiments and evaluated the first-order model candidates that considered the memory of error and generalization across movement directions, here we expanded our list of candidate models that considered the higher-order effects and error-dependent learning rates. We also employed cross-validation to select the leading models. We found that the first-order model with a constant learning rate was the best at predicting learning curves. This model revealed an interaction between the learning and forgetting processes using the direction-specific memory of error. As expected, learning effects were observed at the practiced movement direction on a given trial. Forgetting effects (error increasing) were observed at the unpracticed movement directions with learning effects from generalization from the practiced movement direction. Our study provides insights that guide optimal training using the machine-learning algorithms in areas such as sports coaching, neurorehabilitation, and human-machine interactions.

## Introduction

Motor skill learning requires both movement repetition as well as variation. Repeating the same action provides certainty but practicing different actions can yield better performance through properly assigning credit to relevant factors (Cothros et al., 2006; Berniker and Kording, 2008; Kluzik et al., 2008). However, repetition leads to *bias*, where the more practiced motions are emphasized in learning, and variation allows for *generalization* across related motor actions. Consequently, single task repetition may lead to poor transfer to other motor actions (such as forehand to backhand in tennis) (Atkeson et al., 1997; Schaal and Atkeson, 1998). It is unclear how action repetition and action variation are optimally incorporated during learning, and it may be that a model is needed to best describe this process.

Experimental training schedules can mediate how much repetition and variation of actions are exercised that determine the extent of learning (Shea and Kohl, 1990; Memmert, 2006). While a c*onstant* training schedule that requires repetition of a single motor action may lead to better performance during the training phase, transfer to novel motor actions is limited. On the other hand, a *variable* training schedule that provides exposure to several different motor actions can lead to an increase in the transfer capability (Wulf and Schmidt, 1997; Lee et al., 2016). Researchers have further shown that randomized-variable training (vs. blocked) enhances skill retention and generalization because it demands greater cognitive effort and ensues higher activation of neural structures involved in the planning and execution of motor skills (Lage et al., 2015). However, during the randomized training, different motor actions are practiced consecutively across trials that can have either constructive or destructive interference (Sing and Smith, 2010), and the same motor action might not be repeated until several other motor actions are completed. Iterative updates of an internal model (Kawato, 1990, 1999; Jordan and Rumelhart, 1991) from the non-consecutive repetitive movements with interferences across consecutive trials have not been well-studied.

Error produced during practice plays an important role in the iterative update of motor commands. Motor learning is often characterized as a first-order linear process, where the amount of change in performance is proportional to error using a constant learning rate (Kawato et al., 1987; Wolpert and Kawato, 1998; Thoroughman and Shadmehr, 2000; Scheidt et al., 2001). However, recent studies have shown that the proportional amount of learning from error decreases and saturates toward large error magnitudes, indicating an error-dependent learning rate (Robinson et al., 2003; Fine and Thoroughman, 2007; Wei and Kording, 2009; Marko et al., 2012; Herzfeld et al., 2014). Such non-linearity was observed during learning through the blocked practice of single motor action (e.g., single movement direction). Models that can describe learning in a more real-life scenario through randomized practice (across different movement directions) remain to be identified.

For non-consecutive repetitive movements (i.e., different actions each trial), the motor system might either keep a memory specific to each action, or generalize what was learned to related actions, or both. For memory, some modeling approaches have characterized the learning system as a higher-order linear process, consisting of fast and slow processes (Smith et al., 2006; Kording et al., 2007; Joiner and Smith, 2008; Lee and Schweighofer, 2009). The fast process learns from rapid trial-to-trial changes in error, and the slow processes learn from error components that are consistent across multiple trials. It is then foreseeable that such a learning system could incorporate past movement performance across non-consecutive repetition of actions using the higher-order process. As the repetitive practice is often discrete, a history of errors from at least the past n-trials is necessary to fully realize the nth-order linear process of learning. However, the memory capacity (n) can limit the number of past experiences that are utilized for updating the internal model. It remains to be identified to what extent the error history is involved in motor learning.

The memory may also be susceptible to dynamic changes during the trial gaps between the repetition of the same movement. Phenomena associated with memory dynamics such as consolidation, interference, saving, washout, and forgetting have been widely reported that influence the extent of learning and retention (Brashers-Krug et al., 1996; Caithness et al., 2004; Krakauer et al., 2005; Smith et al., 2006; Krakauer, 2009; Huang et al., 2011). Our earlier work (Parmar and Patton, 2019) tested memory update using the momentum method (Rutishauser, 1959; Polyak, 1964; Sutskever et al., 2013) vs. static memory during the trials when movements specific to the memory are not practiced, and we found that the momentum method was superior at predicting changes in reaching movements during learning visuomotor skills.

Beyond memory for each movement direction, learning can also generalize to solve the non-consecutive action problem. Generalization of learned skill has been observed across arms, across sensory systems, and across arm configurations (Shadmehr and Mussa-Ivaldi, 1994; Dizio and Lackner, 1995; Krakauer et al., 2000; Shadmehr and Moussavi, 2000; Malfait et al., 2002; Criscimagna-Hemminger et al., 2003; Parmar et al., 2015; Bittmann and Patton, 2017). Researchers have found that the internal model generalizes to nearby movement directions or arm configurations (Gandolfo et al., 1996; Sainburg et al., 1999; Thoroughman and Shadmehr, 2000; Donchin et al., 2003; Witney and Wolpert, 2003; Malfait et al., 2005; Berniker et al., 2014). The breadth of generalization is often not known across various tasks, movement directions, and other contexts. Donchin et al. (2003) first defined a model that accounted for the important aspects of generalization of skill learning across movement directions through trial-to-trial performances during the force-field learning. However, they did not test for the momentum effects (memory dynamics) in addition to the generalization effects.

In summary, we must consider higher-order models with multiple time constants, error-dependent learning rates, generalization, and momentum effects. In a preliminary study (Parmar and Patton, 2018), we evaluated the first-order model candidates that considered the memory of error with momentum and generalization. Here, we expanded our list of candidate models that include the higher-order effects and error-dependent learning rates and employed cross-validation to select the leading models. We used a specialized dataset from our earlier study (Parmar and Patton, 2018, 2019) that randomized movement directions across trials, augmented visual error (Patton et al., 2013), and blocked eight distinct visuomotor distortions to obtain multiple learning curves from each subject. We specifically tested how the nervous system updates the initial ballistic launch to reaching targets (first submovement) in response to a visual error. By fitting various models to the learning data, we tested whether the motor system exhibits: (1) constant vs. error-dependent learning rates, (2) first-order vs. higher-order processes, and (3) generalization across movement directions.

## Materials and Methods

### Subjects

Fifteen right-handed healthy human subjects (6M, 9F; within 21–40 years of age) were recruited in this study. These subjects had no history of neurological, shoulder, or elbow disorders. We excluded subjects with ambidexterity. The experimental procedures involving human subjects described in this study were approved by the Institutional Review Boards at Northwestern University (IRB ID: STU00202566) and University of Illinois at Chicago (Protocol # 2016-0911). Human subjects were recruited in this study after obtaining written informed consent approved by the local ethics committee.

### Experimental Setup

Subjects sat in front of a manipulandum robot, and we strapped their right arm wrist to the end effector, the handle, using a wrist brace. We supported their elbow using a multi-link arm support [Basic Mobile Arm Support (MAS) Kit by North Coast] so that their arm movements were planar. The arm support had three degrees of rotational freedom in a plane and had significantly lower inertia compared to a human arm.

The manipulandum was a lightweight, low friction, two degrees of rotational freedom robot (Fayé, 1986). The manipulandum was designed for clinical and neurorehabilitation research applications and was configured through impedance control for safe, stable, and compliant operation. Two low-inertia direct current torque motors (PMI Corp. model JR24M4CH, Kolmorgen Motion Technologies, Commack, NY) were mounted on the base of the robot and were connected independently to each joint using a parallelogram arrangement. The robot's handle position measurements (400 Hz) were taken using two optical encoders (model 25/054-NB17-TA-PPA-QARIS, Teledyne Gurley, Troy, NY, USA).

An opaque, rectangular white screen was positioned horizontally above the robot to block subjects' view of their arm when interacting with the robot. The subjects were seated such that this horizontal screen did not allow them to lean forward. In addition, they were instructed not to lean sideways. We used a 40-inch display to show the position of the handle (as a cursor) and visual targets for reaching. The display was mounted directly above the robot, approximately centered at eye level. We calibrated the display to represent the absolute spatial workspace of the handle (from −44.5 to +44.5 cm in x and from 22 to 72 cm in y of the robot coordinate).

### Experimental Procedure

We seated the subjects such that their right shoulder was directly in front of the robot's shoulder. We measured the subjects' upper arm length, forearm length, and the distance between their shoulder and the robot's shoulder. These measurements were used for the visuomotor distortions (explained below).

Each subject was instructed to move the handle of the robot to bring the cursor toward the center of a target by making a single, quick straight-line reach. The cursor was 2.5 mm diameter white circle, and the targets were 4.5 cm yellow “+” signs. The reaching task included moving the cursor from one target to the next (target-to-target reaching). Only the destination target for a trial was shown at a time on the display. Targets were placed at the vertices of a 15 cm equilateral triangle. The visual location of these targets on the display was fixed for all phases and all non-linear visuomotor distortions. For some positions and orientations of this target set, their corresponding locations in the movement space (the robot's handle space) can be outside the subject's reachable workspace due to a non-linear visuomotor transformation. Therefore, we performed an optimization that minimized the distance between the target locations in the movement space and the center of a typical subject's reachable workspace and maximized the difference in hand-to-vision distortion among all eight non-linear visuomotor distortions. Using the result of this optimization, the center of the triangle was placed in the robot coordinate at 0 cm in x and 47 cm in y and was oriented 75.243 degrees from the x-axis. We confirmed that this set of visual targets was adequately within the reachable workspace for all recruited subjects.

For each target reach, the *initial launch of movement* was detected based on distance and speed thresholds (>1 cm away from the start position and > 20 cm/s), and the end of the initial launch of movement was detected based on speed threshold (<5 cm/s). All the thresholds were calculated in the cursor space. Once the end of the initial launch of movement was detected, the “+” sign for a target was changed to the 4.5 cm “x” sign. At this point, the trial was marked completed, and if the subjects had missed the target (>0.5 cm away from the target position), they were asked to navigate the cursor to the target to begin the next trial. Throughout this navigation phase of the movement, the cursor trace for the initial launch of movement was displayed.

In addition, we provided average-speed feedback of the initial launch of movement using a visual bar at the bottom of the display. The subjects were instructed to match their launch speed with a reference speed bar (indicating 30 cm/s), which was drawn underneath their speed feedback bar. The initial average launch speed within 24–36 cm/s was marked satisfactory with a change in target color and speed feedback bar color to green. Blue represented slower speeds, and red represented faster speeds.

The cursor position was removed for some trials (*no-vision trials*) during the entire initial launch of the movement. Also, the cursor trace for the initial launch of movement was not displayed for these no-vision trials.

**Figure 1:**
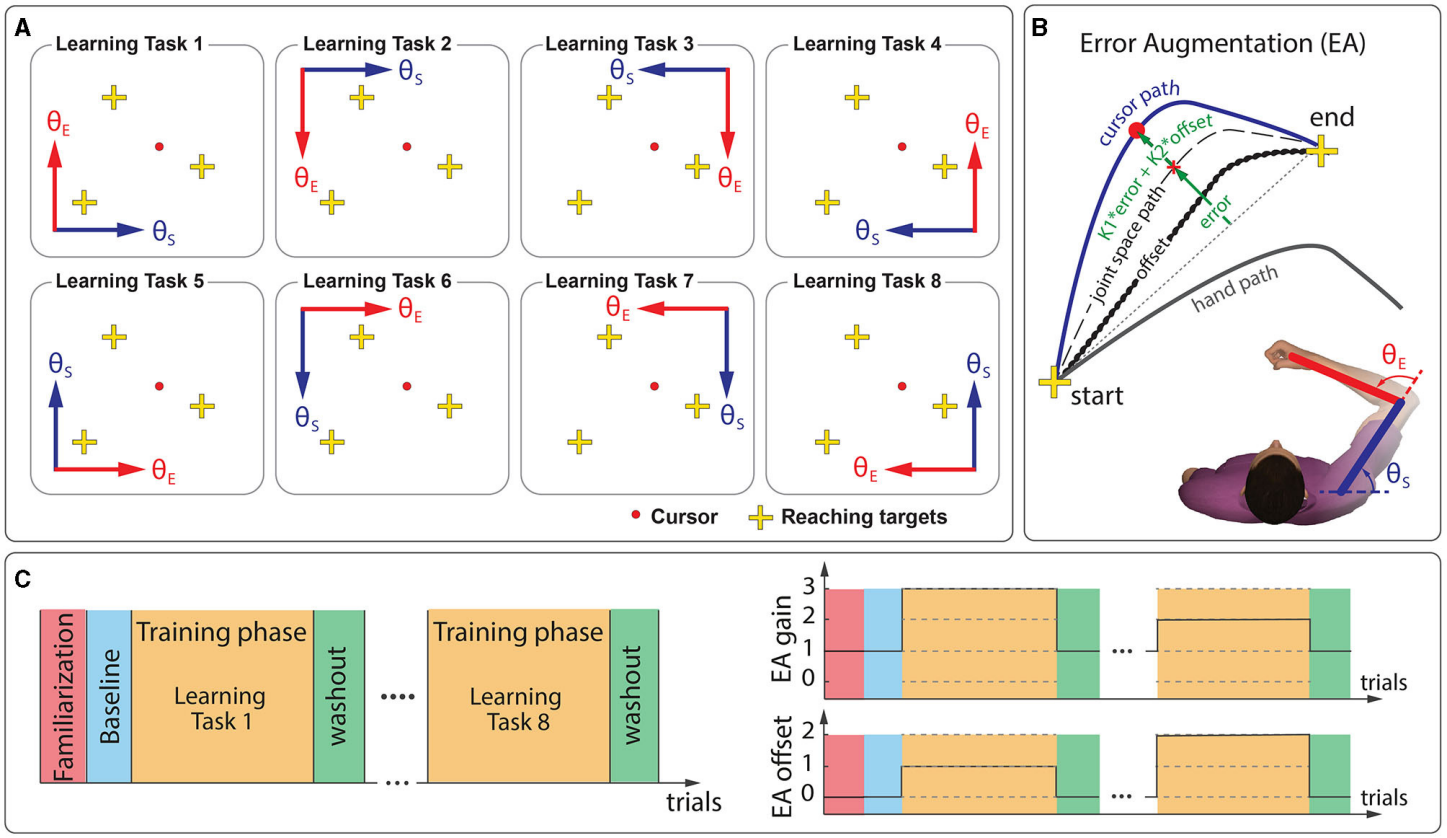
Experimental setup. Participants reached for visual targets using a cursor by moving the handle of a manipulandum robot. **(A)** Their learning tasks were to adapt to various visuomotor distortions by practicing target reaching. **(B)** Subjects received augmented visual feedback based on error (error-augmentation or EA) during the training phases. We administered two types of EA: augmentation of the current level of error (EA-gain; K1; levels = 0, 1, 2, 3) and augmentation of the error-offset (EA-offset; K2; levels = 0, 1, 2). The error-offset was the error subjects produced during initial exposure to the learning tasks. **(C)** The experiment consisted of several distinct phases that included a brief familiarization phase (30 vision trials), a baseline phase (30 no-vision trials), and eight blocks of training phases (250 trials each). Each training phase was followed by a washout phase (30 vision trials). Subjects reached for visual targets at randomly chosen 6 movement directions across trials throughout the experiment. Five subjects received the normal error-feedback (EA{gain 1, offset 0}) throughout the whole experiment, and ten other subjects received randomly chosen EA{gain, offset} feedback from the given levels for each learning task (represented as dashed lines within the training phases).

During the learning phases, cursor represented the subject's shoulder angle vs. elbow angle, instead of hand position. Similar adaptation to a non-linear visuomotor transformation was studied previously by Flanagan and Rao (1995). Here, we could either map shoulder angle along the horizontal dimension (+x) of the display and elbow angle along the vertical dimension (+y) of the display or vice versa. We could also multiply shoulder and elbow angles with −1 to show their mirror transformations on the display. As shown in Figure 1A, we changed how shoulder and elbow angles mapped on the display. This resulted in eight distinct non-linear visuomotor transformations (learning tasks).

Shoulder and elbow angles were calculated using inverse kinematics (Spong et al., 2006):


(1) $$\begin{array}{ll} D & \overset{def}{=}\frac{x^{2}+y^{2}-L_{1}^{2}-L_{2}^{2}}{2\;L_{1}\;L_{2}}, \end{array}$$

(2) $$\begin{array}{l} \theta_{E}=\text{ta}{\text{n}}^{-1}\left(\frac{\sqrt{1-D^{2}}}{D}\right), \end{array}$$

(3) $$\begin{array}{l} \theta_{S}=\text{ta}{\text{n}}^{-1}\left(\frac{y}{x}\right)-\text{ta}{\text{n}}^{-1}\left(\frac{L_{2}\sin\theta_{E}}{L_{1}+L_{2}\cos\theta_{E}}\right)\;, \end{array}$$

where *x* and *y* are the subjects' right arm wrist positions in their shoulder-centered coordinate system. *L*_1_ and *L*_2_ are the subjects' upper and forearm lengths in meters. θ_*S*_ and θ_*E*_ are the subjects' shoulder and elbow joint angles, respectively. Distance between the subjects' shoulder and the robot's shoulder was used to calculate *x* and *y* in the subjects' shoulder-centered coordinate system from the robot's handle position using a homogeneous transformation. For visual calibration, 11 degrees of joint angles represented 5 cm on the display. Furthermore, θ_*S*_ = 2° and θ_*E*_ = 111° were fixed at the center of the display (at 0 cm in x and 47 cm in y of the robot coordinate).

The experiment consisted of several distinct phases (Figure 1C). After a brief familiarization (30 vision trials in the null visual environment, where 1 cm of handle movement represented 1 cm of cursor movement), we assessed baseline performance with 30 no-vision trials in the null environment. Next, all subjects experienced eight different learning tasks (250 trials each): task # 1, 2, 3, 4, 5, 6, 7, and 8 in order. Each learning phase was followed by a washout phase (30 trials). All subjects experienced the same random sequence of reaching targets chosen from the set of 6 movement directions (3 targets). Furthermore, the number of different movement directions experienced by the subjects within a phase was nearly equal. The subjects experienced no-vision trials intermittently (one in four; never two in succession; randomly distributed throughout the total number of trials).

From the first six trials of each learning phase, we assessed initial exposure to a new visuomotor distortion. Each of these six trials was a new direction of reach and a no-vision trial. In order to record a proper initial exposure to the visual distortion, we needed the cursor to be at a specific start position for a particular direction of reach when the visual distortion was applied. We pre-calculated corresponding start positions in the movement space for all directions of reach and for all eight visual distortions. These start positions represented locations of the visual target set in the movement space the subjects would have reached had they learned the visuomotor distortions. At the beginning of these trials, subjects navigated the cursor in the null visual environment to a visual target presented at the location of a start position in the movement space. Once they were at the start, the cursor jumped to its corresponding location in the joint space. Such discontinuity in cursor feedback was unavoidable due to the nature of the visuomotor distortions, and any continuous feedback during the transition from movement space to visual distortion space would have provided learning opportunity for subjects. Next, the target at the start position was blanked, and the target at the end position for that trial was presented. The subjects were then asked to reach. Once the end of the initial launch of movement was detected for these initial exposure trials, we immediately changed the cursor feedback to the null visual environment so that subjects can navigate to start position of a next trial. These initial exposure trials were the no-vision trials and did not provide visual feedback about the visuomotor distortions, and thus we assume in our analysis that the subjects did not learn from these trials.

Our main goal was to identify the best model structure that relates updates to the initial ballistic launch to reaching targets in response to a visual error. Here, we used the error-augmentation (EA) paradigm (Patton et al., 2013), which augments visual feedback based on error. The error-augmentation excites the nervous system with a broad range of sensory stimuli than typical normal feedback, allowing us to better model the learning process (Narendra and Annaswamy, 1987). To span a broad range of errors, we administered two types of EA (Figure 1B): augmentation of the current level of error (EA-gain; gain levels = 0, 1, 2, 3) and augmentation of the error-offset (EA-offset; gain levels = 0, 1, 2). Wide variety of EA gain and EA offset conditions enabled us to acquire a rich set of learning responses to visual error than a typical motor learning experiment. The cursor position was augmented using the following equation when error-augmentation was applied:


(4) $$\begin{array}{l} \left[\begin{array}{c} x_{augmented\;cursor}\\ y_{augmented\;cursor} \end{array}\right]\\ \;=\left[\begin{array}{c} x_{ideal}\\ y_{ideal} \end{array}\right]+EA-gain*\left[\begin{array}{c} x_{cursor}-x_{ideal}\\ y_{cursor}-y_{ideal} \end{array}\right]\\ \;+EA-offset*\left[\begin{array}{c} x_{IE}-x_{ideal}\\ y_{IE}-y_{ideal} \end{array}\right] \end{array}$$

where (*x*_*ideal*_, *y*_*ideal*_) is the straight-line path between starting position and target position for movements, and (*x*_*cursor*_, *y*_*cursor*_) is the cursor position on the display, representing subjects' shoulder and elbow angles (θ_*S*_ and θ_*E*_). The final term in the Equation (4) is the error-offset, which was simply a playback of error (distance from ideal straight-line) that the subjects produced during the initial exposure (*IE*) to the learning tasks. The error-offset provided persistent error throughout training to allow greater error reduction and even *overcompensation* where there is continued learning beyond the goal (Patton et al., 2013). All x-y positions in the Equation (4) were indexed using the path-length from staring position of movements because time samples of x-y positions for the ideal straight-line path were unknown. The error-augmentation was applied only during the initial launch of the movement. Once the end of the initial launch of movement was detected, the augmented error-feedback transitioned smoothly to the normal error-feedback, EA{gain 1, offset 0}, within 50 ms using a sigmoid.

On the error-feedback gain space, there were 12 possible combinations of EA{gain, offset}, which we denote as a set of *EA Coordinates*. Five subjects received normal error-feedback, EA{gain 1, offset 0} for all eight learning tasks. Other ten subjects received the error-augmentation, where EA Coordinate was randomly chosen from the set per learning task (EA coordinate was never repeated within a subject and EA{gain 1, offset 0} was excluded). We repeated the same random order of EA coordinate per task for every two subjects. Note that the order in which the learning task was presented was kept the same across subjects, and only the order in which the error-augmentation applied was randomized.

### Data Analysis

The handle positions were transformed to the objective space (visual display space) by first converting them to the joint angles and then to their corresponding positions on the display {*joint space trajectory*; [*x*_*cursor*_(*t*), *y*_*cursor*_(*t*)]; using the calibration methods provided above}. For the data analysis, we did not further transform these joint space trajectories using EA-gain and EA-offset, which the subjects saw during the experiment.

Next, we calculated the maximum L2-norm error between time samples of the first submovement of the joint space trajectory and an ideal straight-line path between the start and goal positions. The first submovement was identified by the first speed hump > 14 cm/s speed that drops more than 2 cm/s on either side. The onset and termination of the first submovement were marked at the lowest speed <5 cm/s. The straight-line path was the minimum jerk profile (Flash and Hogan, 1985). The initial launch of movement (the first submovement) could be to the left or to the right of the ideal straight-line. Thus, we assigned a positive sign to the error if the initial launch direction (calculated at the maximum error) for the performed movement was toward the same side as the initial exposure movement and a negative sign for the opposite case. We used the following equations to calculate the maximum L2-norm error:


(5) $$\begin{array}{l} {\left\|error(t)\right\|}_{2}=\;\sqrt{{\left(x_{cursor}\left(t\right)-x_{ideal}\left(t\right)\right)}^{2}+{\left(y_{cursor}\left(t\right)-y_{ideal}\left(t\right)\right)}^{2}},\; \end{array}$$

(6) $$\begin{array}{l} \max\;error\;=error\;Sign\;*\;\max\left({\left\|error(t)\right\|}_{2}\right), \end{array}$$

We also approximated number of segmented movements within each trial (throughout whole movement from start to end target) by measuring number of prominent humps in speed profiles. Each speed hump was identified by finding peak (local maxima) > 14 cm/s that drops more than 2 cm/s on either side.

### Estimation of Learning From the Initial Launch

**Figure 2:**
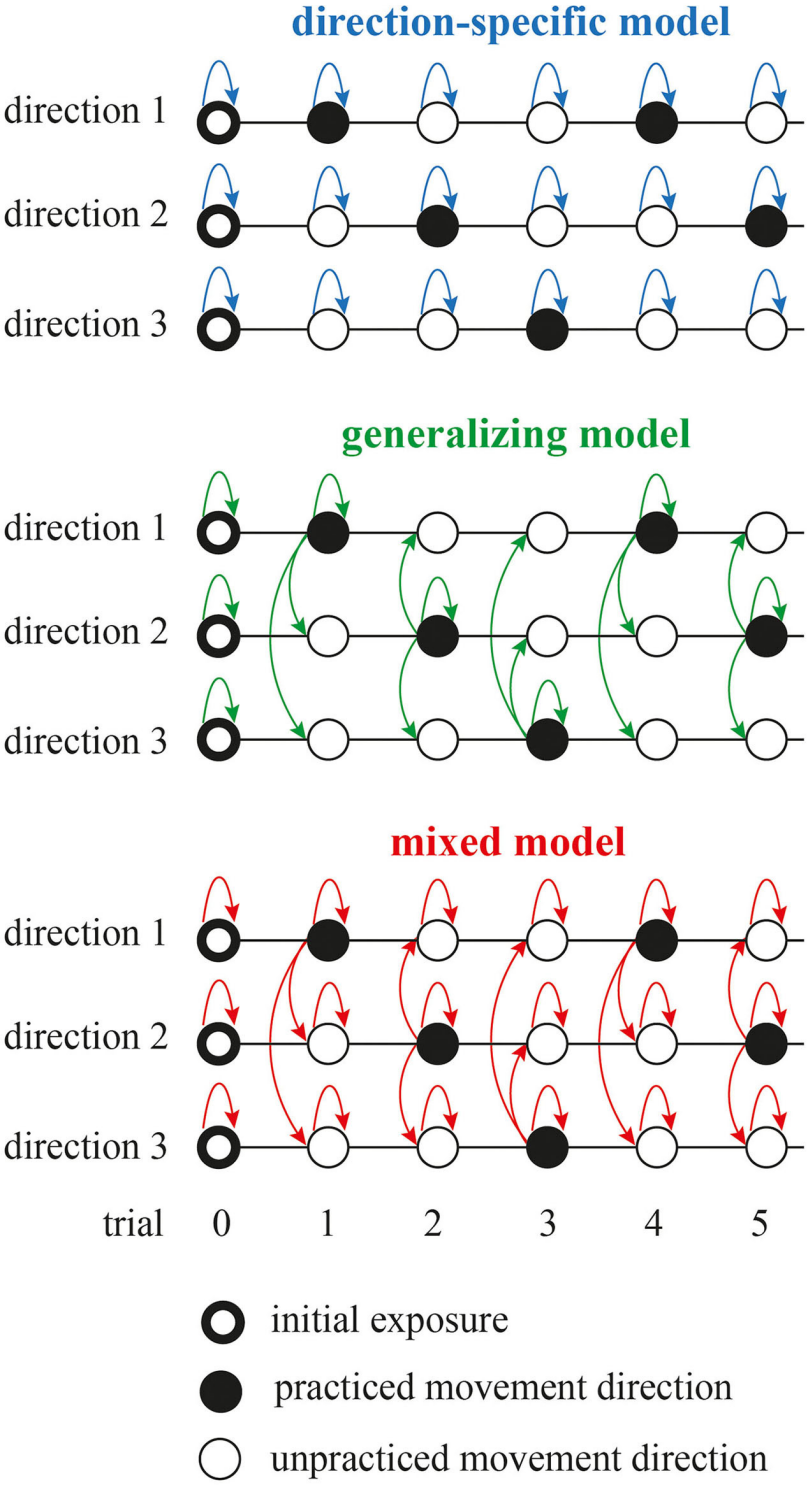
Three model schemes to assess how the initial ballistic launch to the target is shaped by the visually perceived error. The direction-specific model assumes that the learning is independent for each movement direction. The generalizing model assumes that the learning for all movement directions is dependent on the visual error perceived at the practiced movement direction. The mixed model is the weighted sum of previous two models. Dark and white circles depict practiced and unpracticed movement directions, respectively. Ring-shaped markers at trial zero represent initial condition for the models that was based initial exposure to the learning task. Arrows represent update to the model state based on the model structure. Note that the direction-specific model allowed for updates to the model state during the trial gaps between the practiced trials using momentum method. Also note that only three movement directions are shown here for brevity, but there were six movement directions in the experiment.

We assessed how the initial ballistic launch to the target (the first submovement of trial) is shaped by the visually perceived error. We tested three model schemes (Figure 2): direction-specific, generalizing, and mixed. While the preliminary study (Parmar and Patton, 2018) introduced these different modeling schemes, it only examined linear and affine model structures without any cross-validation. Furthermore, to evaluate learning, the preliminary study used average error as the metric that seemed to bias the metric toward zero error (root-mean-squared error from the first 150 ms of movement). Here, we used maximum L2-norm error between time samples of the first submovement of the joint space trajectory and the straight-line path between start and goal positions.

In the direction-specific model, learning is independent for each movement direction. Here, we assessed how the visually perceived error at trial *n* (ê_*n*_) affects the movement error at trial *n* + 1 (*e*_*n*+ 1_):


(7) $$\begin{array}{l} e_{n+1,d}=e_{n,d}+f_{d}\left({ê}_{n,d}\right). \end{array}$$

Here, the visually perceived error was estimated as


(8) $$\begin{array}{l} {ê}_{n,d}=EA-gain\;*\;e_{n,d}+EA-offset\;*\;e_{0,d}, \end{array}$$

where *EA*-*gain*, *EA*-*offset* were augmentation levels that the subjects experienced, and the error offset, *e*_0_, was the error during the initial exposure. Subscript *d* represents movement direction. This direction-specific model was updated every trial regardless of whether the movement direction was experienced. This is to allow for any dynamic changes during the trial gaps between the repetition of the same movement direction. For the practiced movement direction, *EA*-*gain* and *EA*-*offset* were set to the values subjects experienced, and for the unpracticed movement direction, *EA*-*gain* and *EA*-*offset* were set to the normal feedback condition (EA{gain 1, offset 0}).

The generalizing model assumes that the learning for all movement directions is dependent on the visual error perceived at the practiced movement direction. We define the generalized perceived error as:


(9) $$\begin{array}{l} {ĝ}_{n,d}=W\left(\theta_{d,p}\right)\;*\;{ê}_{n,p}, \end{array}$$

where *W* is generalization weight between two movement directions with separation of θ degrees. Subscript *p* is the practiced movement direction. *W* was constrained between −1 and 1. In this experiment, there were six movement directions and possible angular separations among them include θ = [−120, −60, 0, 60, 120, 180]. *W*(0) was fixed at 1 and others were free parameters in the optimization. Here, we used Equation (7) where we had *f*_*d*_(ĝ_*n,d*_) instead of *f*_*d*_(ê_*n,d*_). This generalizing model is similar to the model first defined by Donchin et al. (2003) that accounted for the important aspects of generalization of skill learning across movement directions through trial-to-trial performances during the force-field learning.

Finally, the mixed model included the memory dynamics in addition to the generalization effects. We assessed how the perceived error (ê_*n*_) and generalized perceived error (ĝ_*n*_) at trial *n* affect the movement error at trial *n* + 1 (*e*_*n*+1_) across movement directions:


(10) $$\begin{array}{l} e_{n+1,d}=e_{n,d}+f_{d}\left({ê}_{n,d}\right)+Z_{d}{ĝ}_{n,d}. \end{array}$$

We first tested whether the learning rate associated with the amount of learning from the visually perceived error was constant vs. error magnitude dependent. The following first-order models captured the effects associated with constant learning rates:


(11) $$\begin{array}{l} \text{Linear}:\left(1.1\text{L}\right)\;f_{d}\left({ê}_{n,d}\right)=B_{d}{ê}_{n,d} \end{array}$$

(12) $$\begin{array}{l} \text{Affine}:\left(1.1\right)\;f_{d}\left({ê}_{n,d}\right)=A_{d}+\;B_{d}{ê}_{n,d} \end{array}$$

We then considered learning rate as linear and quadratic functions of error, which led us to the following quadratic and cubic polynomial functions of inter-trial change:


(13) $$\begin{array}{l} \text{Quadratic}:\left(1.2\right)\;f_{d}\left({ê}_{n,d}\right)=A_{d}+\left(B_{d}+C_{d}{ê}_{n,d}\right){ê}_{n,d}\\ \;=A_{d}+B_{d}{ê}_{n,d}+C_{d}{ê}_{n,d}^{2} \end{array}$$

(14) $$\begin{array}{l} \text{Cubic}:\left(1.3\right)\;f_{d}\left({ê}_{n,d}\right)=A_{d}+\left(B_{d}+C_{d}{ê}_{n,d}+D_{d}{ê}_{n,d}^{2}\right){ê}_{n,d}\\ \;=A_{d}+B_{d}{ê}_{n,d}+C_{d}{ê}_{n,d}^{2}+D_{d}{ê}_{n,d}^{3} \end{array}$$

We also considered Gaussian-weighted learning rate:


(15) $$\begin{array}{l} \text{Gaussian}:\left(1.1\text{G}\right)\;f_{d}\left({ê}_{n,d}\right)=A_{d}+\;B_{d}\exp\left(-{\left(\frac{{ê}_{n,d}}{H_{d}}\right)}^{2}\right){ê}_{n,d} \end{array}$$

We also considered linear higher-order processes that included error history from the past 2 to 8 trials:


(16) $$\begin{array}{l} \text{kth-order}\left(k>1\right):\left(k.1\right)\;f_{d}\left({ê}_{n,d}\right)=A_{d}+\;B_{1,d}{ê}_{n,d}+\ldots \\ \;+\;B_{k,d}{ê}_{n-k+1,d} \end{array}$$

We also tested higher-order process with quadratic non-linearity:


(17) $$\begin{array}{l} \text{kth-order}\left(k>1\right):\left(k.2\right)\;f_{d}\left({ê}_{n,d}\right)=A_{d}+\;B_{1,d}{ê}_{n,d}+\ldots \\ \;+\;B_{k,d}{ê}_{n-k+1,d}+C_{d}{ê}_{n,d}^{2}. \end{array}$$

We relabeled the coefficients *B, C, D* to *Z, Y, X*, respectively, to indicate learning from the generalized error, Equation (9). We assigned each model a unique set of identifiers as labeled in **Figure 4** and Supplementary Figures 1, 3, 4. The first identifier indicates whether the model is direction-specific, generalizing, or mixed (ds, g, m), the second identifier indicates whether the model is first-order, second-order, or higher-order process (1, 2, etc.), and the third identifier indicates whether the model has non-linearity using first-order, second-order, or third-order polynomials (1, 2, 3). 1L represents linear polynomial without an offset term, and 1G represents Gaussian-weighted learning rate.

Because of the sparse observation data across trials for each movement direction, we could not perform a typical regression analysis by correlating movement errors from one trial to the next. Here we used a simulation-based approach to fitting models. Given the subjects' initial exposure errors (*e*_0_), the model structure and the model parameters, the model iteratively generated a sequence of movement errors across all trials for each movement direction (using the model generated movement error from a trial to generate movement error for the next trial). Then, using non-linear least squares (1,000-fold GlobalSearch method with interior-point algorithm, MathWorks MATLAB R2017b), we tuned the model parameters to best match the generated movement errors to the observed movement errors from the subjects at the practiced movement direction trials. During the initialization of the simulation for the higher-order model structures, the model states required for the trials earlier than 0th trial (initial exposure trial) were set to zero. We used the movement error from both the vision and no-vision trials of the learning phases, and the visually perceived error (Equation 8) was set to zero for the no-vision trials. We removed the observations where EA-gain was zero because this condition did not allow any variation in the visually perceived error to identify its relationship to the inter-trial change in error.

During the simulation for a movement direction, the model allowed update to the direction-specific model state (movement error) on the trials that were not experienced by the subjects for that particular movement direction. This update is similar to the momentum method used with the gradient descent algorithm during optimization routines where a state change from a previous step is repeated for the current step (Rutishauser, 1959; Polyak, 1964; Sutskever et al., 2013). Our earlier work (Parmar and Patton, 2019) tested the momentum-based update rule against a static update rule (no change to the direction-specific model state) during the trial gaps when a particular movement direction is not practiced, and we found that the momentum method was superior at predicting changes in reaching movements during the learning of visuomotor skills.

The number of free parameters for each model for fitting each learning phase data were as follows. For the direction-specific model, the number of free parameters were equal to the number of model structure parameters times six movement directions: ds.1.1G = 18, ds.1.3 = 24, ds.1.2 = 18, ds.1.1 = 12, ds.1.1L = 6, ds.2.1 = 18, ds.3.1 = 24, ds.4.1 = 30, ds.5.1 = 36, ds.6.1 = 42, ds.7.1 = 48, ds.8.1 = 54, ds.2.2 = 24, ds.3.2 = 30, ds.4.2 = 36. For the generalizing model, the number of free parameters were equal to the number of model structure parameters times six movement directions plus five generalization weights: g.1.3 = 29, g.1.2 = 23, g.1.1 = 17, g.1.1L = 11, g.2.1 = 23, g.3.1 = 29. For the mixed model, the number of free parameters were equal to the number of model structure parameters times six movement directions plus five generalization weights: m.1.3 = 35, m.1.2 = 29, m.1.1 = 23, m.1.1L = 17, m.2.1 = 29, m.3.1 = 35, m.4.1 = 41.

### Statistical Analysis

We fitted the above models per movement direction per learning task and per subject. During the model regressions, we used all 250 trials of the training phases. However, during the analysis of test set partitions for the cross-validation, we only used the first 50 trials of training phases. This is because the learning measurements across trials are often decaying transient signals, from which the learning signals are prominently detectable above a noise level in the earlier epoch of training phases. As the transient signals decay, the component of noise becomes more dominant in the steady state. Therefore, when predicting the test set partition dataset using the best fit models, the earlier observations of the training phases provide greater distinction among the model structures. Also note that noise in the steady state would not affect the model regressions because the least-squares optimization would find the best tuning for the model parameters across all trials.

We calculated the coefficient of determination (*R*^2^ and $R_{adj}^{2}$) for each regression fit (102 estimates from 8 learning tasks x 15 subjects minus learning tasks where EA-gain = 0). In order to mitigate overfitting, these models were exhaustively cross-validated across subjects and across tasks within subject. For the cross-validation across subjects, we partitioned 15 subjects into the training and test sets (4/5 and 1/5, respectively; 455 total independent partitions). For the cross-validation across tasks within subject, we partitioned 8 learning tasks into the training and test sets (4/5 and 1/5, respectively; 28 total independent partitions for each subject; 28^*^15 total independent partitions across all subjects). We exhaustively tested all possible permutations of partitioning the training and test sets, and we assessed the accuracy of the average and median models from the training set onto the test set using the root-mean-square error (RMSE). For each test set partition, the distribution of RMSE was not normal, and thus we calculated the maximum likelihood estimate (MLE) of RMSE from their kernel density estimates. Such an exhaustive cross-validation analysis enabled us to avoid overfitting.

We performed pairwise comparisons among the models for the MLE of RMSE from all test set partitions using the left-tailed Wilcoxon signed rank test with 0.01 alpha level, corrected using Bonferroni method. All reported *p*-values are multiplied with the appropriate correction factors. We then computed the model score as the number of times a particular model had significantly lower RMSE compared to other models minus the number of times other models had significantly lower RMSE compared to a particular model. For the best cross-validated models, we performed the sign test (alpha 0.05) to identify the model parameters that have non-zero median across all subjects and learning tasks. We also performed Kruskal-Wallis one-way ANOVA tests (alpha 0.05) with learning task as a major factor and movement direction as a major factor for each model parameters to identify learning task dependent effects on the learning trends.

## Results

Typical of any reaching study, all subjects reached for the visual targets in straight-lines with nearly symmetric and smooth velocity profiles during the baseline phase (no-vision trials) with the absolute error of 7.2 ± 3.1 *mm* (mean±95% confidence interval). The subjects also reached in straight lines toward the targets by the end of the washout phases (last six vision trials) with an absolute error of 4.5 ± 1.8 *mm*.

**Figure 3:**
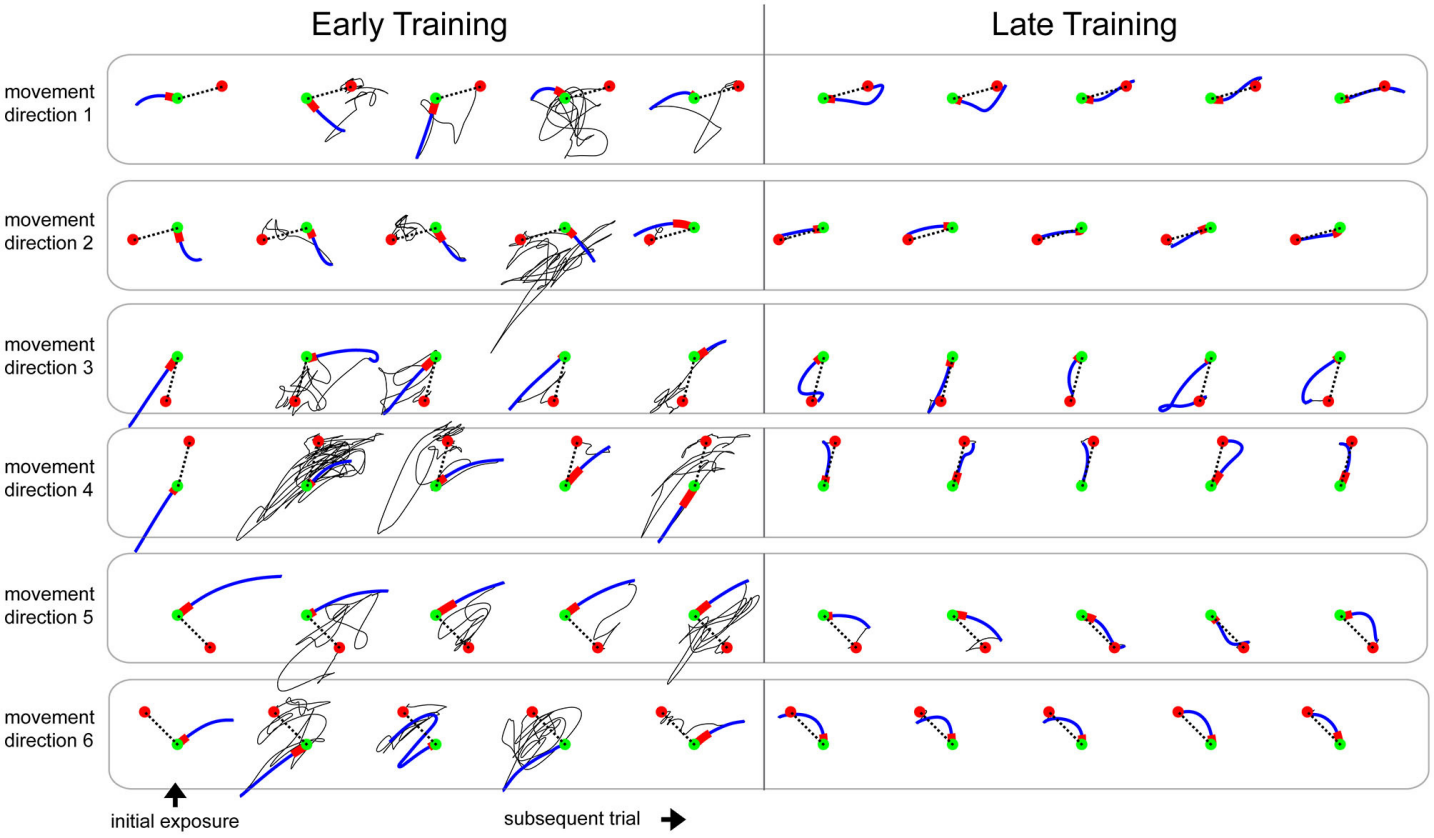
Representative examples of movement paths from a subject practicing the learning task 3 with EA{gain 1, offset 0}. Green and red circles represent the start and target positions for the movements (15 cm apart). The dotted black line is the ideal straight line. Blue segment is the initial launch of the movement, and red segment is the first 150 ms of the initial launch. Solid Black line is the feedback correction phase of the movement where the subject navigated to the target after the initial launch. Note that the feedback correction phase is highly random during the early training and is greatly reduced and systematic during the late training.

During the initial exposure to the learning tasks (first 6 trials), the subjects produced a substantial error (absolute error of 20.19 ± 0.92 *cm*, no-vision trials), which were characteristically distinct based on the learning tasks and movement directions. As depicted by the examples in Figure 3 and Supplementary Figure 6, reaching movements early in the training phase were highly variable across subsequent trials, and the corrective actions after the initial launch were seemingly random. Note in these examples that the direction of the initial launch varied more during the early training but became consistent by the end of the training. As seen in an example learning curve from a subject (Supplementary Figure 5), the movement errors from the initial launches to the targets reduced as training progressed. Also, the length of the corrective actions became smaller and systematic toward the end of the training phases as the subjects practiced. We measured 16.8 ± 6 segmented movements (approximated by the number of prominent humps in speed profiles; averaged across the subjects who received normal feedback) during early training (first 5 trials) and 3.5 ± 0.6 segmented movements during late training (last 5 trials). We measured 2.5 ± 0.6 segmented movements during the baseline phase.

We exhaustively cross-validated several model structures that assessed how the initial launch to the targets (first submovement of the trials) are shaped by visually perceived error. The model structures included the learning rates that were either constant, error magnitude dependent (linear and quadratic functions of error), or Gaussian-weighted. The models also used the error history from past 2 to 8 trials. Furthermore, the models considered generalization across movement directions. The regression statistics are shown in Supplementary Figure 1.

**Figure 4:**
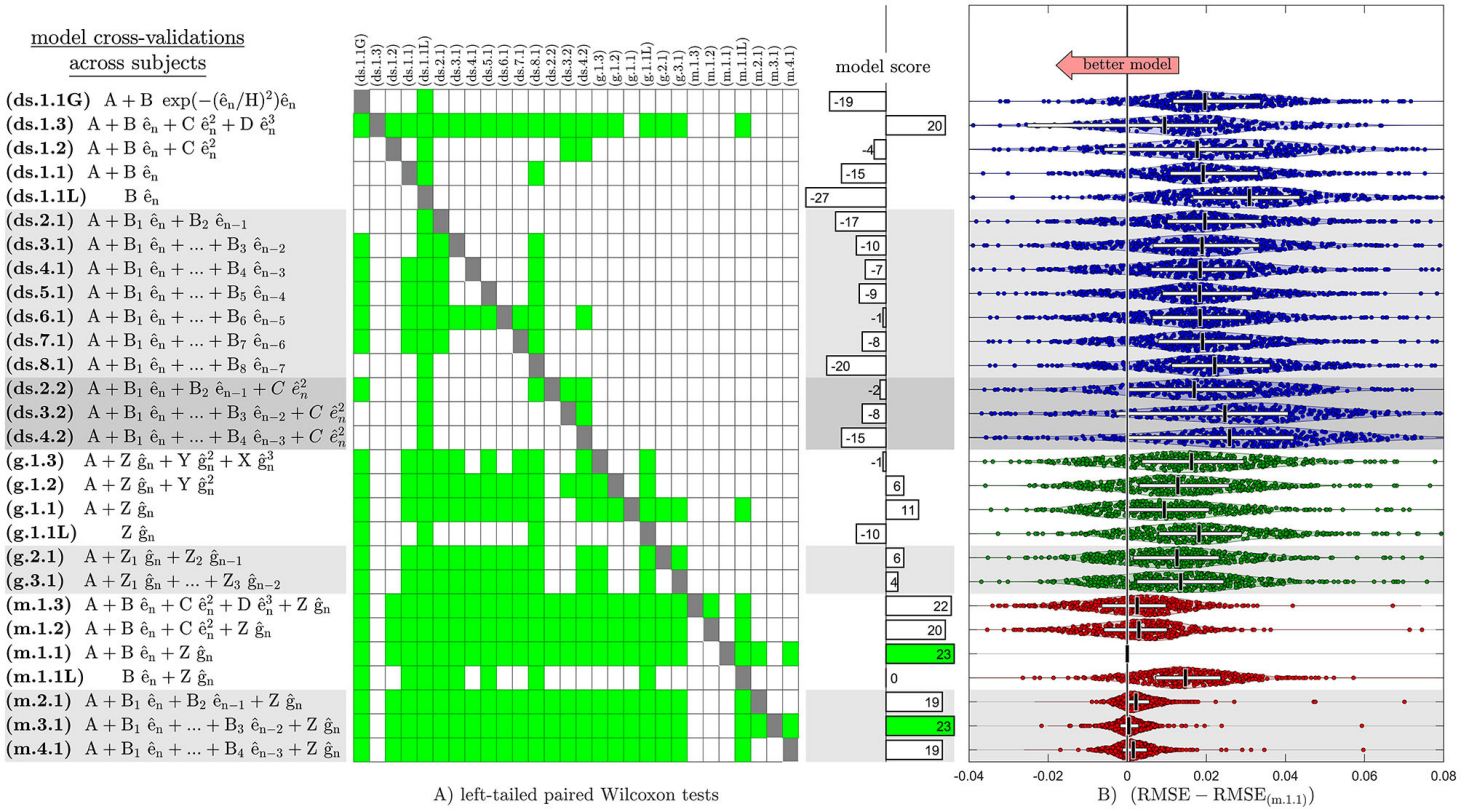
Accuracy between model predictions and observations during the cross-validation across subjects, indicating the superiority of the first-order mixed model (m.1.1). **(A)** Left-tailed paired Wilcoxon tests are shown on a matrix, where a green square indicates significantly lower RMSE in the cross-validation test set partitions (*p* < 0.01; with Bonferroni correction; toward lower RMSE) for the model listed along its row compared to the model listed along its column. No statistical tests were performed along the diagonal of the matrix as indicated by dark gray squares. The histogram next to the matrix shows the model score as the number of times a particular model had significantly lower RMSE compare to other models minus the number of times other models had significantly lower RMSE compare to a particular model (number of green squares across a model's row number of green squares across a model's column). **(B)** Each dot represents a paired difference between its respective model's and the first-order mixed model's (m.1.1) MLE of RMSE for a cross-validation test set partition. Horizontal white bars and vertical black bars represent inter-quartile range and median of the paired differences of RMSE across all cross-validation test set partitions. Shaded regions show kernel density estimates for the distribution. The first- and third-order mixed models with constant learning rates (m.1.1 and m.3.1) had significantly lowest RMSE (*p* < 0.01) and performed equally best in the cross-validation test set partitions across subjects. The first-order mixed model (m.1.1) was clearly the winner since it had the lowest number of model parameters.

When cross-validating across subjects, we found that the median models from the training set predicted systematic changes in the movement errors from the test set significantly better than the average models (*p* < 0.01; with Bonferroni correction). The cross-validations identified two models that had the significantly lowest RMSE (*p* < 0.01; with Bonferroni correction) in the test-set partitions across subjects (Figure 4; Supplementary Figure 3). These models were the first- and third-order mixed models with constant learning rates (m.1.1 and m.3.1), which had RMSE significantly lower than 23 out of 28 total model candidates. The first-order mixed model with learning rates that were quadratic functions of error magnitude (m.1.3) was the second-best that had RMSE significantly lower than 22 out of 28 total model structures. Among these, the first-order mixed model (m.1.1) had the lowest number of model parameters.

While the cross-validation analysis across subjects identified the model structure (i.e., m.1.1) that is consistently able to predict the learning trends for new subjects, we wanted to further test whether the same model structure is also able to predict the learning trends for new learning tasks within subject. When cross-validating across tasks within subject, we again found that the median models from the training set predicted systematic changes in the movement errors from the test set significantly better than the average models (*p* < 0.01; with Bonferroni correction). This cross-validation across tasks identified the first-order direction-specific model with constant learning rates (ds.1.1) that had the significantly lowest RMSE (*p* < 0.01, with Bonferroni correction) in the test-set partitions (Supplementary Figure 4). This model (ds.1.1) had RMSE significantly lower than 22 out of 28 total model candidates.

**Figure 5:**
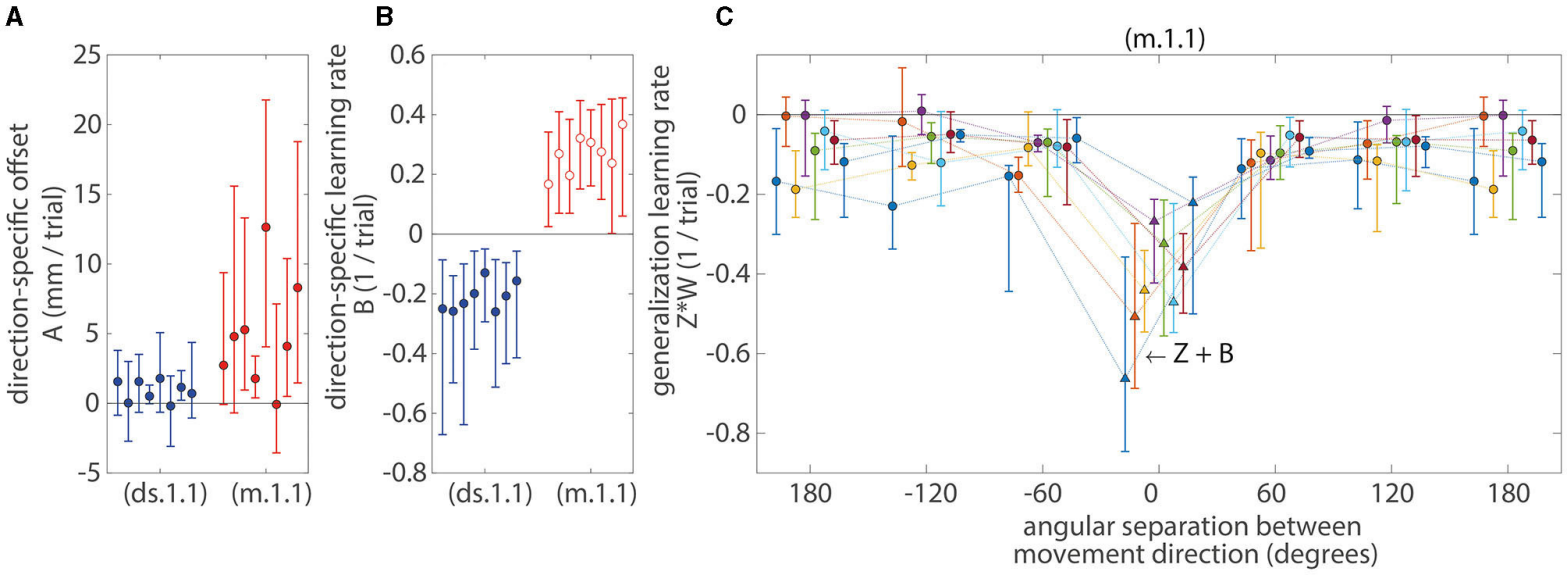
Median parameter values for the first-order affine models. **(A)** The model offsets (*A* value). **(B)** The learning rates associated with the direction-specific error (B value). **(C)** The effective learning rates associated with generalization across movement directions, Z*W(θ), see Equation (9) and (10). Markers (circles and triangles) indicate median values and error bars indicate IQR across all subjects and directions. A set of eight error bars (in all subplots and lines in **C**) for each model represents eight learning tasks. The offset parameters (*A* value) for both the direction-specific (ds.1.1) and mixed models (m.1.1) were significantly positive (*p* < 0.05; sign-test) and were also learning task dependent (*p* < 0.05; Kruskal-Wallis test). The learning rates associated with the direction-specific error (*B* value) for the direction-specific model (ds.1.1) were significantly negative (*p* < 0.05; sign-test) and were also learning task dependent (*p* < 0.05; Kruskal-Wallis test). When memory for a movement direction could update for the mixed model (m.1.1) during the trial when that particular movement direction was practiced (i.e., *W* = 1 and θ = 0), the effective learning rates (*Z* + *B* value; triangles) were significantly negative (*p* < 0.05; sign-test). However, when memory for a movement direction could update using the momentum method for the mixed model (m.1.1) during the trial when that particular movement direction was not practiced (i.e., *W* ≠ 1 and θ ≠ 0; open red circle), the learning rates (*B* value) were significantly positive (*p* < 0.05; sign-test). The presence of these positive learning rates indicates that there were error increasing processes (e.g., forgetting or unlearning). For the mixed models (m.1.1), the effective learning rates associated with the generalized error (Z*W) were more negative at zero degrees of angular separation between movement directions but became less negative as the angular separation increased to 60 degrees or more. These learning rates were symmetric across positive and negative (clockwise and counterclockwise) angular distances. All effective learning rates associated with the generalized error (Z*W) were significantly negative (*p* < 0.05; sign-test) and were learning task dependent at 120 and 180 degrees of angular distances (*p* < 0.05; Kruskal-Wallis test).

The effective learning rates for the first-order affine models are shown in Figure 5. The offset parameters (*A* value) for both the direction-specific (ds.1.1) and mixed models (m.1.1) were significantly positive (*p* < 0.05; sign-test across subjects and learning tasks) and were also learning task dependent (*p* < 0.05; Kruskal-Wallis test with learning task as a factor). Furthermore, the offset parameters (*A* value) for the direction-specific model (ds.1.1) were movement direction dependent (*p* < 0.05; Kruskal-Wallis test with movement direction as a factor). The learning rates associated with the direction-specific error (*B* value) for the direction-specific model (ds.1.1) were significantly negative (*p* < 0.05; sign-test across subjects and learning tasks) and were also learning task dependent (*p* < 0.05; Kruskal-Wallis test with learning task as a factor).

For the mixed model (m.1.1), there were instances when variance was shared between the learning rates (*B* and *Z* values) that were associated with the direction-specific error (ê) and with the generalized error (ĝ). When memory for a movement direction could update for the mixed model (m.1.1) during the trial when that particular movement direction was practiced [i.e., *W* = 1 and θ = 0, see Equations (9) and (10)], the effective learning rates (*Z* + *B* value) were significantly negative (*p* < 0.05; sign-test across subjects and learning tasks). However, when memory for a movement direction could update using the momentum method for the mixed model (m.1.1) during the trial when that particular movement direction was not practiced (i.e., *W* ≠ 1 and θ ≠ 0), the learning rates (*B* value) were significantly positive (*p* < 0.05; sign-test across subjects and learning tasks). The presence of these positive learning rates indicates that there were error increasing processes, such as forgetting or unlearning.

For the mixed models (m.1.1), the effective learning rates associated with the generalized error (Z^*^W) were more negative at zero degrees of angular separation between movement directions but became less negative as the angular separation increased to 60 degrees or more. These learning rates were symmetric across positive and negative (clockwise and counterclockwise) angular distances. All effective learning rates associated with the generalized error (Z^*^W) were significantly negative (*p* < 0.05; sign-test across subjects and learning tasks), and these learning rates were learning task dependent at 120 and 180 degrees of angular distances (*p* < 0.05; Kruskal-Wallis test with learning task as a factor performed at each angular separation).

## Discussion

We investigated how the neuromotor system may incorporate randomly perceived visual error across movement directions to update its trial-to-trial initial launch to reaching targets. We used computational models to test whether the learning rates were constant, error magnitude dependent, or Gaussian-weighted. We also tested whether error history from past 2 to 8 trials are used and whether errors are generalized across movement directions. Our cross-validation analysis identified the first-order mixed model with constant learning rates (m.1.1) that is consistently able to predict the learning trends for new subjects across all learning tasks (Figure 4; Supplementary Figure 3). We further tested whether the same model structure is also able to predict the learning trends for new learning tasks within each subject, and this cross-validation analysis identified the first-order direction-specific model with constant learning rates (ds.1.1; Supplementary Figure 4). The mixed model structure (m.1.1) allowed for the generalization of learning across movement direction, whereas the direction-specific model (ds.1.1) did not. This difference in the model structures indicated that the generalization of learned skill across movement direction was not consistent across different leaning tasks. We did in fact find that the subjects learned different generalization patterns for at least some of the learning tasks (Figure 5).

The first-order mixed model (m.1.1) revealed an interaction between the learning and forgetting processes. This model exhibited learning at the practiced movement direction on a given trial as expected (negative *Z*+*B* value at zero degrees in Figure 5C). The forgetting process (error increasing positive learning rates; *B* value in Figure 5B) was observed at the unpracticed movement directions (with learning effects from generalization from the practiced movement direction, Z^*^W). Donchin et al. (2003) first defined a model that accounted for the generalization of skill learning across movement directions through trial-to-trial performances, but their model did not include any dynamics associated with memory. Our list of candidate model structures also included the generalizing models (e.g., model g.1.1) that were similar to theirs (Donchin et al., 2003). However, our cross-validation analysis indicated that both the direction-specific memory dynamics (decay of learning effects or unlearning) and the generalization should be accounted for (e.g., using model m.1.1) to predict trial-to-trial changes in the initial launch to reaching targets during learning. Trial-to-trial unlearning has been observed earlier (Scheidt et al., 2000, 2001; Thoroughman and Shadmehr, 2000), and researchers have posited that there might be a distinct neural system that allows for rapid unlearning (Smith et al., 2006; Hirashima and Nozaki, 2012). Here, our first-order mixed model (m.1.1) incorporates learning, forgetting and generalization effects across movement directions during randomized training.

Variety of results about the shape of the generalization curves that span across movement directions have been observed. Some studies found asymmetric transfer of learning to the clockwise and counterclockwise side from the trained direction (Darainy et al., 2009; Gonzalez Castro et al., 2011). A recent study further found inconsistent asymmetries that depended on the trained movement directions (Rezazadeh and Berniker, 2019). Other studies did not find such asymmetries (Mattar and Ostry, 2007). In this study, while we also failed to detect asymmetric generalization of the learning across movement directions, the pattern of generalization was learning task dependent. The determinant factors for the shape of the generalization curves that span across movement directions are not well-understood. Some studies report that limb stiffness can account for the varying generalization curves (Darainy et al., 2009; Rezazadeh and Berniker, 2019). Limb stiffness can change either due to change in reach direction or due to newly learned motor commands (Burdet et al., 2001; Franklin et al., 2003). Here, it could have been that limb stiffness varied across at least some of the eight learning tasks of this study. However, our models did not account for limb stiffness. It remains to be seen whether the mixed model (m.1.1) that accounts for limb stiffness can predict learning trends for a new learning task (within subject) better than the direction-specific model (ds.1.1).

Consistent with prior work (Gandolfo et al., 1996; Sainburg et al., 1999; Donchin et al., 2003; Witney and Wolpert, 2003; Malfait et al., 2005; Berniker et al., 2014), we also found that the transfer of the learning effects was local (within 60 degrees) from where movement errors were perceived. This narrowly generalizing learning provides evidence for a modular structure of the motor learning system (Ghahramani and Wolpert, 1997; Wolpert and Kawato, 1998; Flanagan et al., 1999; Kawato, 1999). Some researchers estimated the shape of the generalization function to be bimodal (Donchin et al., 2003; Wainscott et al., 2005), where they estimated larger effects of generalization at 0 and 180 degrees than intermediate degrees of angular distance between movement directions. Here, we did not find the larger effects of generalization at 180 degrees than intermediate angles.

We found weak indications for the sub-linear error-dependent learning rates (using model m.1.3). This has been shown in other studies that proportionate amount of learning from error at higher error magnitude is reduced (Robinson et al., 2003; Fine and Thoroughman, 2007; Wei and Kording, 2009; Marko et al., 2012). Recently, a theoretical framework was proposed to describe how the memory of error experienced over multiple sessions (or blocks) may give rise to the error-dependent learning rate (Herzfeld et al., 2014). In their study, the learning task was one dimensional (one target) and simple, i.e., the perturbations were a linear function of hand position or velocity, where the subjects presumably could have a so-called *informative prior* (Bolstad and Curran, 2017) that resulted in the systematic corrective movements and guided learning. So, for example, when forces pushed the hand to the left, subjects knew how to readily correct by moving to the right. Furthermore, their theoretical framework used error-dependent learning rate to explain the phenomenon of “savings” and “meta-learning,” where prior experience can facilitate re-adaptation (Kojima et al., 2004; Braun et al., 2009; Malone et al., 2011; Turnham et al., 2012; Sarwary et al., 2013; Herzfeld et al., 2014). In our experiment, the learning tasks were non-linear hand-to-vision mappings where the subjects seemed to have an *uninformative* prior that did not prescribe proper actions, resulting in almost random corrective search in the early training (Figure 3; Supplementary Figure 6). Furthermore, while the subjects had eight blocks of training in our study, each learning task was novel (possibly interfering); subjects never experienced the same error across different training blocks. Thus, our weak detection for an error-dependent learning rate should also be considered as a model candidate to be tested on a different dataset.

When the motor system is lacking in prior knowledge of what perceptual changes accompany the motor outputs, movements are likely to be more variable as a consequence (Figure 3; Supplementary Figure 6). Moreover, EA-gain enlarges not only the magnitude of error but also the variability associated with the error. While such variation is argued to be both useful for a breadth of experience, it might also be destructive due to perceived uncertainty (Takiyama et al., 2015). Researchers have shown that the brain can actively regulate motor variability (Mandelblat-Cerf et al., 2009), and these highly variable movements have the potential to facilitate error-based motor learning (Wu et al., 2014). However, the heightened motor variability leads to inconsistent feedback, and how the error in this type of feedback is evaluated by the motor system to update its feedforward plan remained unexplained. Our results (using model m.3.1) provide the possibility of a higher-order learning process that can incorporate such variable errors from the history of past errors.

Variability in experiences is not to be confused with unpredictability in the environment. Variability in feedback can be due to random environmental disturbances, natural physiological (signal-dependent) noise (Schmidt et al., 1979; Harris and Wolpert, 1998; Jones et al., 2002; van Beers et al., 2002; Todorov, 2004; Osborne et al., 2005; O'Sullivan et al., 2009; Shmuelof et al., 2012), and other active sources (Mandelblat-Cerf et al., 2009; Wu et al., 2014). Learning is less effective unless perceived errors have some form of consistency across multiple trials. Thus, the motor system must have mechanisms to properly assign credit to the sources of error (Berniker and Kording, 2008; Wei and Kording, 2009) to keep track of consistent error. Researchers have shown that the motor system can reduce the learning rate in a rapidly changing environment (Herzfeld et al., 2014), and the motor system is most likely to learn only from the latest error in the stochastic non-stationary environment, rather than from error history (Scheidt et al., 2001). In our study, because sources of variation came more from the intention and enlarged due to EA, learning may depend on error history. The nature of the variability of error as seen by the subjects was different in our study compared to other studies (Scheidt et al., 2001; Herzfeld et al., 2014). In their study, the perturbations were random or rapidly changing trial-to-trial, so the same two motor outputs can result in different feedback. In our study, however, visual distortions did not change, and the same two motor outputs produced the same feedback. Furthermore, any variability in the visual feedback our subjects perceived was the augmented version of their motor variability under the EA paradigm, and there was no external source of the random disturbance.

It would be interesting to further investigate whether the current results about the motor learning obtained for the right-handed individuals with the right arm training is also corroborated with the left-arm training. However, handedness is an important topic in the control of arm movements, and researchers have reported lateralization of motor performance and inter-limb asymmetries in goal-directed control of arm movements (Sainburg and Kalakanis, 2000; Wang and Sainburg, 2007). Under the dynamic-dominance hypothesis, researchers have demonstrated that the control of right arm for the right-handed individual is optimized for limb coordination or dynamics while the control of non-dominant left arm is optimized for the end-point positional accuracy (Sainburg, 2002, 2005; Yadav and Sainburg, 2014). Such hemispheric specialization of motor control can influence adaptation of movement skills (McGrath and Kantak, 2016). The role of handedness during adaptation of movement skills through randomized training schedule across multiple movement directions remains to be further studied.

While the model structure identified in the current study (model m.1.1) matched previous results (Parmar and Patton, 2018), its parameter values differed. Differences from previous work here could be because of methodological changes. The current study used a different error metric (maximum error from the whole initial launch to the target vs. root-mean-square error from the first 150 ms of movement we used in Parmar and Patton, 2018). Furthermore, the current study presented median parameter values since median models performed superior under cross-validation. Whereas the preliminary results of the parameter values were based on average models, which were not cross-validated. In this study, we used cross-validation to evaluate the quality of and select the best model. Cross-validation helps prevent idiosyncratic fitting and accommodate latent variables.

Many confounding factors might have led to poor model fits (Supplementary Figure 1). For example, we found only about 41.11, 39.49, 39.14, and 29.39% of variance accounted (*R*^2^ MLE) by the models that performed best under cross-validations (m.1.3, m.1.1, m.3.1, and ds.1.1, respectively). The poor *R*^2^ may be attributed to a low SNR, arising from the error-augmentation experimental conditions, the difficult learning tasks, and the randomized experiment design. For the first-order mixed model (m.1.1), we found a negative correlation between *R*^2^ and standard deviation of residual error (Supplementary Figure 2). This indicated that low SNR (approximated as high standard deviation of residual error) deteriorated the model fit quality (*R*^2^). Furthermore, in a separate analysis, we found that the standard deviation of residual error increased with EA-gain. Such an effect was expected because EA-gain condition amplifies the variability associated with the movement error. Also, the visuomotor distortions used in this study were non-linear and highly difficult to learn. This can be seen in the movement traces of the early training trials (Figure 3; Supplementary Figure 6) where the corrective actions post the initial launch were seemingly random and lengthy. Other studies that used this type of visuomotor distortion (Flanagan and Rao, 1995) also reported the similar results for the early training trials. Also, the experiment design in this study used randomized trial sequence across movement directions. Such sequences can have either constructive or destructive interference (Sing and Smith, 2010) in learning across consecutive trials. Also, it is well-known that exposure to conflicting visuomotor rotations or force fields is very hard if not impossible to learn. While here the different visuomotor distortions were not intermixed, they followed closely (with a short washout after each). Thus, the previous exposure might have interfered with learning in the next exposure. However, since the order in which the different visuomotor distortions presented was kept the same for all subjects, any such interference may have been comparable across subjects. Furthermore, the models tested in this study only used the initial launch movements and did not account for any learning that might have taken place during the feedback correction phase of movements when the subjects navigated to the target after the initial launch. All these factors may have led to a low SNR. We changed our error metric from the preliminary analysis (maximum error from the whole initial launch to the target vs. root-mean-square error from the first 150 ms of the movement that we used in Parmar and Patton, 2018), but the model *R*^2^ remained similar. Despite poor *R*^2^, the cross-validation analysis provides indications about the model structures of learning that remain consistent across subjects.

There are several different methods for model selections, but the forward stepwise regression was the best suitable method for our dataset. To select the best model structure, we incrementally added a term to the model and evaluated the model to see whether the additional terms yield statistically better prediction error for new subjects or new learning tasks (cross-validation test sets). In this ground up approach, we fitted candidate model structures for each subject and each learning task separately within a training set partition, and we evaluated the median model on the test set partition. Alternatively, we could have applied machine learning methods (such as LASSO regularization) on our most complex model to select the best predictor terms. However, when using the LASSO method with our dataset, handling of inter-subject variations and intra-subject variations (variations across learning tasks) poses a challenge. Model fitting with LASSO for each subject and each learning task separately may not select the same predictor terms for each case, and thus, deriving some average model out of models with different predictors may not be feasible for the cross-validation. Furthermore, fitting a model with LASSO on the whole training set partition would disregard both inter-subject and intra-subject variations. It remains to be seen whether the LASSO method yields the same results as our ground-up approach.

Our study provides insights into how visually perceived error from sparse repetitive movements across trials while practicing reaching to several different movement directions shape motor learning of visuomotor skills. Randomized-variable training schedules that require practicing several different movement directions consecutively can enhance the acquisition of skills through a robust internal model. This suggests that the machine-learning algorithms used for developing optimal training for neurorehabilitation, sports coaching, and human-machine interactions should include direction-specific specific models with narrow generalization to neighboring movement directions.

## Data Availability Statement

The raw data supporting the conclusions of this article will be made available by the authors, without undue reservation.

## Ethics Statement

The studies involving human participants were reviewed and approved by the Institutional Review Boards at Northwestern University (IRB ID: STU00202566) and University of Illinois at Chicago (Protocol # 2016-0911). The patients/participants provided their written informed consent to participate in this study.

## Author Contributions

PP and JP conceived and designed the experiments and wrote the paper. PP performed the experiments and analyzed the data. Both authors contributed to the article and approved the submitted version.

## Funding

The research reported in this publication was supported by the National Institutes of Health Award Numbers F31-NS100520 and 2R01-NS053606.

## Conflict of Interest

The authors declare that the research was conducted in the absence of any commercial or financial relationships that could be construed as a potential conflict of interest.

## Publisher's Note

All claims expressed in this article are solely those of the authors and do not necessarily represent those of their affiliated organizations, or those of the publisher, the editors and the reviewers. Any product that may be evaluated in this article, or claim that may be made by its manufacturer, is not guaranteed or endorsed by the publisher.

## Supplementary Material

Supplementary Figure 1 Summary of regression statistics using the coefficient of determination (*R*^2^ and $R_{adj}^{2}$). The model structures listed on the left represent the inter-trial change in movement error in response to the visually perceived error during the initial launch of movement. Each colored circle represents its respective statistics for a particular learning task for subjects (102 estimates from 8 learning tasks x 15 subjects minus learning tasks with EA-gain = 0). Shaded colored regions show kernel density estimates for the distribution. Horizontal white bars and vertical black bars represent inter-quartile range and median across all subjects and learning tasks (blue = direction-specific model; green = generalizing model; red = mixed model).

Supplementary Figure 2 Relationship between *R*^2^ and standard deviation of residual error for the first-order mixed model (m.1.1). White circles represent learning tasks for subjects. Shaded colored region represents the kernel density estimate of the data. Red curve represents the linear regression between *R*^2^ and standard deviation of residual error (F-test on the linear regression model against constant model yielded *p* = 2.64^*^10^−8^). Low SNR (approximated as high standard deviation of residual error) deteriorated the fit quality (*R*^2^) for the first-order mixed model (m.1.1).

Supplementary Figure 3 Accuracy between model predictions and observations during the cross-validation across subjects, indicating the superiority of the first-order mixed model (m.1.1). **(A)** Left-tailed paired Wilcoxon tests are shown on a matrix, where a green square indicates significantly lower RMSE in the cross-validation test set partitions (*p* < 0.01; with Bonferroni correction; toward lower RMSE) for the model listed along its row compared to the model listed along its column. No statistical tests were performed along the diagonal of the matrix as indicated by dark gray squares. The histogram next to the matrix shows the model score as the number of times a particular model had significantly lower RMSE compared to other models minus the number of times other models had significantly lower RMSE compared to a particular model (number of green squares across a model's row number of green squares across a model's column). **(B)** Each dot represents the MLE of RMSE for a cross-validation test set partition. Horizontal white bars and vertical black bars represent inter-quartile range and median across all subjects, directions, and learning tasks. Shaded regions show kernel density estimates for the RMSE distribution. The first- and third-order mixed models with constant learning rates (m.1.1 and m.3.1) had significantly lowest RMSE (*p* < 0.01) and performed equally best in the cross-validation test set partitions across subjects. The first-order mixed model (m.1.1) was clearly the winner since it had the lowest number of model parameters.

Supplementary Figure 4 Accuracy between model predictions and observations during the cross-validation across tasks, indicating the superiority of the first-order direction-specific model (ds.1.1). **(A)** Left-tailed paired Wilcoxon tests are shown on a matrix, where a green square indicates significantly lower RMSE in the cross-validation test set partitions (*p* < 0.01; with Bonferroni correction; toward lower RMSE) for the model listed along its row compared to the model listed along its column. No statistical tests were performed along the diagonal of the matrix as indicated by dark gray squares. The histogram next to the matrix shows the model score as the number of times a particular model had significantly lower RMSE compared to other models minus the number of times other models had significantly lower RMSE compared to a particular model (number of green squares across a model's row number of green squares across a model's column). **(B)** Each dot represents the MLE of RMSE for a cross-validation test set partition. Horizontal white bars and vertical black bars represent inter-quartile range and median across all subjects, directions, and learning tasks. Shaded regions show kernel density estimates for the RMSE distribution. The first-order direction-specific model with constant learning rates (ds.1.1) had significantly lowest RMSE (*p* < 0.01) in the cross-validation test set partitions across tasks.

Supplementary Figure 5 An example of the best model fit to learning data from a subject. Circles are the errors performed by a subject during the learning task 4 with EA{gain 1, offset 0}, and red curve is the fit of the affine mixed model (m.1.1). This richness of behavior, such as the oscillation about zero, is possible due to the multiple factors, a signed error metric, and iterative incremental learning considered in the model.

Supplementary Figure 6 Representative examples of movement paths from a subject practicing the learning task 3 with EA{gain 1,offset 1}. Green and red circles represent the start and target positions for the movements (15 cm apart). The dotted black line is the ideal straight line. Blue segment is the initial launch of the movement, and red segment is the first 150 ms of the initial launch. Solid Black line is the feedback correction phase of the movement where the subject navigated to the target after the initial launch. Note that the feedback correction phase is highly random during the early training and is greatly reduced and systematic during the late training.

## References

[B1] Atkeson C. G. Moore A. W. Schaal S. (1997). Locally weighted learning for control, in Lazy Learning ed. Aha D. W. Dordrecht: Springer, 75–113. 10.1007/978-94-017-2053-3_3

[B2] Berniker M. Franklin D. W. Flanagan J. R. Wolpert D. M. Kording K. (2014). Motor learning of novel dynamics is not represented in a single global coordinate system: evaluation of mixed coordinate representations and local learning. J. Neurophysiol. 111, 1165–1182. 10.1152/jn.00493.2013 24353296 PMC3949315

[B3] Berniker M. Kording K. (2008). Estimating the sources of motor errors for adaptation and generalization. Nat. Neurosci. 11, 1454–1461. 10.1038/nn.2229 19011624 PMC2707921

[B4] Bittmann M. F. Patton J. L. (2017). Forces that supplement visuomotor learning: a “sensory crossover” experiment. IEEE Trans. Neural Syst. Rehabil. Eng. 25, 1109–1116. 10.1109/TNSRE.2016.2613443 28113982 PMC5644020

[B5] Bolstad W. M. Curran J. M. (2017). Introduction to Bayesian Statistics. Hoboken, New Jersey: Wiley.

[B6] Brashers-Krug T. Shadmehr R. Bizzi E. (1996). Consolidation in human motor memory. Nature 382, 252–255. 10.1038/382252a0 8717039

[B7] Braun D. A. Aertsen A. Wolpert D. M. Mehring C. (2009). Learning optimal adaptation strategies in unpredictable motor tasks. J. Neurosci. 29, 6472–6478. 10.1523/JNEUROSCI.3075-08.2009 19458218 PMC2692080

[B8] Burdet E. Osu R. Franklin D. W. Milner T. E. Kawato M. (2001). The central nervous system stabilizes unstable dynamics by learning optimal impedance. Nature 414, 446–449. 10.1038/35106566 11719805

[B9] Caithness G. Osu R. Bays P. Chase H. Klassen J. Kawato M.. (2004). Failure to consolidate the consolidation theory of learning for sensorimotor adaptation tasks. J. Neurosci. 24, 8662–8671. 10.1523/JNEUROSCI.2214-04.2004 15470131 PMC6729970

[B10] Cothros N. Wong J. Gribble P. (2006). Are there distinct neural representations of object and limb dynamics? Exp. Brain Res. 173, 689–697. 10.1007/s00221-006-0411-0 16525798

[B11] Criscimagna-Hemminger S. E. Donchin O. Gazzaniga M. S. Shadmehr R. (2003). Learned dynamics of reaching movements generalize from dominant to nondominant arm. J. Neurophysiol. 89, 168–176. 10.1152/jn.00622.2002 12522169

[B12] Darainy M. Mattar A. A. G. Ostry D. J. (2009). Effects of human arm impedance on dynamics learning and generalization. J. Neurophysiol. 101, 3158–3168. 10.1152/jn.91336.2008 19357340 PMC2694125

[B13] Dizio P. Lackner J. R. (1995). Motor adaptation to Coriolis force perturbations of reaching movements: endpoint but not trajectory adaptation transfers to the nonexposed arm. J. Neurophysiol. 74, 1787–1792. 10.1152/jn.1995.74.4.1787 8989414

[B14] Donchin O. Francis J. T. Shadmehr R. (2003). Quantifying generalization from trial-by-trial behavior of adaptive systems that learn with basis functions: theory and experiments in human motor control. J. Neurosci. 23, 9032–9045. 10.1523/JNEUROSCI.23-27-09032.2003 14534237 PMC6740843

[B15] Fayé I. C. (1986). An Impedance Controlled Manipulandum for Human Movement Studies. (Master of Science in Mechanical Engineering SM Thesis), Massachusetts Institute of Technology (MIT).

[B16] Fine M. S. Thoroughman K. A. (2007). Trial-by-trial transformation of error into sensorimotor adaptation changes with environmental dynamics. J. Neurophysiol. 98, 1392–1404. 10.1152/jn.00196.2007 17615136

[B17] Flanagan J. R. Nakano E. Imamizu H. Osu R. Yoshioka T. Kawato M. (1999). Composition and decomposition of internal models in motor learning under altered kinematic and dynamic environments. J. Neurosci. 19:RC34. 10.1523/JNEUROSCI.19-20-j0005.1999 10516336 PMC6782771

[B18] Flanagan J. R. Rao A. K. (1995). Trajectory adaptation to a nonlinear visuomotor transformation: evidence of motion planning in visually perceived space. J. Neurophysiol. 74, 2174–2178. 10.1152/jn.1995.74.5.2174 8592205

[B19] Flash T. Hogan N. (1985). The coordination of arm movements: an experimentally confirmed mathematical model. J. Neurosci. 5, 1688–1703. 10.1523/JNEUROSCI.05-07-01688.1985 4020415 PMC6565116

[B20] Franklin D. W. Osu R. Burdet E. Kawato M. Milner T. E. (2003). Adaptation to stable and unstable dynamics achieved by combined impedance control and inverse dynamics model. J. Neurophysiol. 90, 3270–3282. 10.1152/jn.01112.2002 14615432

[B21] Gandolfo F. Mussa-Ivaldi F. A. Bizzi E. (1996). Motor learning by field approximation. Proc. Natl. Acad. Sci. U. S. A. 93, 3843–3846. 10.1073/pnas.93.9.3843 8632977 PMC39446

[B22] Ghahramani Z. Wolpert D. M. (1997). Modular decomposition in visuomotor learning. Nature 386, 392–395. 10.1038/386392a0 9121554

[B23] Gonzalez Castro L. N. Monsen C. B. Smith M. A. (2011). The binding of learning to action in motor adaptation. PLoS Comput. Biol. 7:e1002052. 10.1371/journal.pcbi.1002052 21731476 PMC3121685

[B24] Harris C. M. Wolpert D. M. (1998). Signal-dependent noise determines motor planning. Nature 394, 780–784. 10.1038/29528 9723616

[B25] Herzfeld D. J. Vaswani P. A. Marko M. K. Shadmehr R. (2014). A memory of errors in sensorimotor learning. Science 345, 1349–1353. 10.1126/science.1253138 25123484 PMC4506639

[B26] Hirashima M. Nozaki D. (2012). Learning with slight forgetting optimizes sensorimotor transformation in redundant motor systems. PLoS Comput. Biol. 8:e1002590. 10.1371/journal.pcbi.1002590 22761568 PMC3386159

[B27] Huang V. S. Haith A. Mazzoni P. Krakauer J. W. (2011). Rethinking motor learning and savings in adaptation paradigms: model-free memory for successful actions combines with internal models. Neuron 70, 787–801. 10.1016/j.neuron.2011.04.012 21609832 PMC3134523

[B28] Joiner W. M. Smith M. A. (2008). Long-term retention explained by a model of short-term learning in the adaptive control of reaching. J. Neurophysiol. 100, 2948–2955. 10.1152/jn.90706.2008 18784273 PMC2585394

[B29] Jones K. E. Hamilton A. F. Wolpert D. M. (2002). Sources of signal-dependent noise during isometric force production. J. Neurophysiol. 88, 1533–1544. 10.1152/jn.2002.88.3.1533 12205173

[B30] Jordan M. I. Rumelhart D. E. (1991). Internal world models and supervised learning, in Machine Learning Proceedings 1991, eds Birnbaum L. A Collins G. C (San Francisco, CA: Morgan Kaufmann), 70–74. 10.1016/B978-1-55860-200-7.50018-0

[B31] Kawato M. (1990). Feedback-error-learning neural network for supervised motor learning. Adv. Neural Comput. 6, 365–372. 10.1016/B978-0-444-88400-8.50047-9

[B32] Kawato M. (1999). Internal models for motor control and trajectory planning. Curr. Opin. Neurobiol. 9, 718–727. 10.1016/S0959-4388(99)00028-8 10607637

[B33] Kawato M. Furukawa K. Suzuki R. (1987). A hierarchical neural-network model for control and learning of voluntary movement. Biol. Cybern. 57, 169–185. 10.1007/BF00364149 3676355

[B34] Kluzik J. Diedrichsen J. Shadmehr R. Bastian A. J. (2008). Reach adaptation: what determines whether we learn an internal model of the tool or adapt the model of our arm? J. Neurophysiol. 100, 1455–1464. 10.1152/jn.90334.2008 18596187 PMC2544452

[B35] Kojima Y. Iwamoto Y. Yoshida K. (2004). Memory of learning facilitates saccadic adaptation in the monkey. J. Neurosci. 24, 7531–7539. 10.1523/JNEUROSCI.1741-04.2004 15329400 PMC6729647

[B36] Kording K. P. Tenenbaum J. B. Shadmehr R. (2007). The dynamics of memory as a consequence of optimal adaptation to a changing body. Nat. Neurosci. 10, 779–786. 10.1038/nn1901 17496891 PMC2551734

[B37] Krakauer J. W. (2009). Motor learning and consolidation: the case of visuomotor rotation, in Progress in Motor Control. Advances in Experimental Medicine and Biology (Boston, MA: Springer), 405–421. 10.1007/978-0-387-77064-2_21 PMC2672910 19227512

[B38] Krakauer J. W. Ghez C. Ghilardi M. F. (2005). Adaptation to visuomotor transformations: consolidation, interference, and forgetting. J. Neurosci. 25, 473–478. 10.1523/JNEUROSCI.4218-04.2005 15647491 PMC6725486

[B39] Krakauer J. W. Pine Z. M. Ghilardi M. F. Ghez C. (2000). Learning of visuomotor transformations for vectorial planning of reaching trajectories. J. Neurosci. 20, 8916–8924. 10.1523/JNEUROSCI.20-23-08916.2000 11102502 PMC6773094

[B40] Lage G. M. Ugrinowitsch H. Apolinário-Souza T. Vieira M. M. Albuquerque M. R. Benda R. N. (2015). Repetition and variation in motor practice: a review of neural correlates. Neurosci. Biobehav. Rev. 57, 132–141. 10.1016/j.neubiorev.2015.08.012 26299808

[B41] Lee J. Y. Oh Y. Kim S. S. Scheidt R. A. Schweighofer N. (2016). Optimal schedules in multitask motor learning. Neural Comput. 28, 667–685. 10.1162/NECO_a_00823 26890347 PMC6555556

[B42] Lee J. Y. Schweighofer N. (2009). Dual adaptation supports a parallel architecture of motor memory. J. Neurosci. 29, 10396–10404. 10.1523/JNEUROSCI.1294-09.2009 19692614 PMC2789989

[B43] Malfait N. Gribble P. L. Ostry D. J. (2005). Generalization of motor learning based on multiple field exposures and local adaptation. J. Neurophysiol. 93, 3327–3338. 10.1152/jn.00883.2004 15659531

[B44] Malfait N. Shiller D. M. Ostry D. J. (2002). Transfer of motor learning across arm configurations. J. Neurosci. 22, 9656–9660. 10.1523/JNEUROSCI.22-22-09656.2002 12427820 PMC6757833

[B45] Malone L. A. Vasudevan E. V. Bastian A. J. (2011). Motor adaptation training for faster relearning. J. Neurosci. 31, 15136–15143. 10.1523/JNEUROSCI.1367-11.2011 22016547 PMC3209529

[B46] Mandelblat-Cerf Y. Paz R. Vaadia E. (2009). Trial-to-trial variability of single cells in motor cortices is dynamically modified during visuomotor adaptation. J. Neurosci. 29, 15053–15062. 10.1523/JNEUROSCI.3011-09.2009 19955356 PMC6665974

[B47] Marko M. K. Haith A. M. Harran M. D. Shadmehr R. (2012). Sensitivity to prediction error in reach adaptation. J. Neurophysiol. 108, 1752–1763. 10.1152/jn.00177.2012 22773782 PMC3774589

[B48] Mattar A. A. Ostry D. J. (2007). Modifiability of generalization in dynamics learning. J. Neurophysiol. 98, 3321–3329. 10.1152/jn.00576.2007 17928561

[B49] McGrath R. L. Kantak S. S. (2016). Reduced asymmetry in motor skill learning in left-handed compared to right-handed individuals. Hum. Mov. Sci. 45, 130–141. 10.1016/j.humov.2015.11.012 26638046

[B50] Memmert D. (2006). Long-term effects of type of practice on the learning and transfer of a complex motor skill. Percept. Mot. Skills 103, 912–916. 10.2466/pms.103.3.912-916 17326522

[B51] Narendra K. S. Annaswamy A. M. (1987). Persistent excitation in adaptive systems. Int. J. Control 45, 127–160. 10.1080/00207178708933715

[B52] Osborne L. C. Lisberger S. G. Bialek W. (2005). A sensory source for motor variation. Nature 437, 412–416. 10.1038/nature03961 16163357 PMC2551316

[B53] O'Sullivan I. Burdet E. Diedrichsen J. (2009). Dissociating variability and effort as determinants of coordination. PLoS Comput. Biol. 5:e1000345. 10.1371/journal.pcbi.1000345 19360132 PMC2661023

[B54] Parmar P. N. Huang F. C. Patton J. L. (2015). Evidence of multiple coordinate representations during generalization of motor learning. Exp. Brain Res. 233, 1–13. 10.1007/s00221-014-4034-6 25248844 PMC4289976

[B55] Parmar P. N. Patton J. L. (2018). Models of motor learning generalization, in 2018 40th Annual International Conference of the IEEE Engineering in Medicine and Biology Society (EMBC), IEEE. 10.1109/EMBC.2018.8513182 PMC8767419 30441402

[B56] Parmar P. N. Patton J. L. (2019). Sparse identification of motor learning using proxy process models, in 2019 16th IEEE/RAS-EMBS International Conference on Rehabilitation Robotics (ICORR), IEEE. 10.1109/ICORR.2019.8779423 31374737

[B57] Patton J. L. Wei Y. J. Bajaj P. Scheidt R. A. (2013). Visuomotor learning enhanced by augmenting instantaneous trajectory error feedback during reaching. PLoS ONE 8:e46466. 10.1371/journal.pone.0046466 23382796 PMC3559751

[B58] Polyak B. T. (1964). Some methods of speeding up the convergence of iteration methods. USSR Comput. Math. Math. Phys. 4, 1–17. 10.1016/0041-5553(64)90137-5

[B59] Rezazadeh A. Berniker M. (2019). Force field generalization and the internal representation of motor learning. PLoS ONE 14:e0225002. 10.1371/journal.pone.0225002 31743347 PMC6863527

[B60] Robinson F. R. Noto C. T. Bevans S. E. (2003). Effect of visual error size on saccade adaptation in monkey. J. Neurophysiol. 90, 1235–1244. 10.1152/jn.00656.2002 12711711

[B61] Rutishauser H. (1959). Theory of gradient methods, in Refined Iterative Methods for Computation of the Solution and the Eigenvalues of Self-Adjoint Boundary Value Problems (Springer), 24–49.

[B62] Sainburg R. L. (2002). Evidence for a dynamic-dominance hypothesis of handedness. Exp. Brain Res. 142, 241–258. 10.1007/s00221-001-0913-8 11807578 PMC10710695

[B63] Sainburg R. L. (2005). Handedness: differential specializations for control of trajectory and position. Exerc. Sport Sci. Rev. 33, 206–213. 10.1097/00003677-200510000-00010 16239839 PMC10709818

[B64] Sainburg R. L. Ghez C. Kalakanis D. (1999). Intersegmental dynamics are controlled by sequential anticipatory, error correction, postural mechanisms. J. Neurophysiol. 81, 1045–1056. 10.1152/jn.1999.81.3.1045 10085332 PMC10715731

[B65] Sainburg R. L. Kalakanis D. (2000). Differences in control of limb dynamics during dominant and nondominant arm reaching. J. Neurophysiol. 83, 2661–2675. 10.1152/jn.2000.83.5.2661 10805666 PMC10709817

[B66] Sarwary A. M. Selen L. P. Medendorp W. P. (2013). Vestibular benefits to task savings in motor adaptation. J. Neurophysiol. 110, 1269–1277. 10.1152/jn.00914.2012 23785131 PMC3763157

[B67] Schaal S. Atkeson C. G. (1998). Constructive incremental learning from only local information. Neural Comput. 10, 2047–2084. 10.1162/089976698300016963 9804671

[B68] Scheidt R. A. Dingwell J. B. Mussa-Ivaldi F. A. (2001). Learning to move amid uncertainty. J. Neurophysiol. 86, 971–985. 10.1152/jn.2001.86.2.971 11495965

[B69] Scheidt R. A. Reinkensmeyer D. J. Conditt M. A. Rymer W. Z. Mussa-Ivaldi F. A. (2000). Persistence of motor adaptation during constrained, multi-joint, arm movements. J. Neurophysiol. 84, 853–862. 10.1152/jn.2000.84.2.853 10938312

[B70] Schmidt R. A. Zelaznik H. Hawkins B. Frank J. S. Quinn J. T. Jr. (1979). Motor-output variability: a theory for the accuracy of rapid motor acts. Psychol. Rev. 47, 415–451. 10.1037/0033-295X.86.5.415 504536

[B71] Shadmehr R. Moussavi Z. M. (2000). Spatial generalization from learning dynamics of reaching movements. J. Neurosci. 20, 7807–7815. 10.1523/JNEUROSCI.20-20-07807.2000 11027245 PMC6772893

[B72] Shadmehr R. Mussa-Ivaldi F. A. (1994). Adaptive representation of dynamics during learning of a motor task. J Neurosci 14, 3208–3224. 10.1523/JNEUROSCI.14-05-03208.1994 8182467 PMC6577492

[B73] Shea C. H. Kohl R. M. (1990). Specificity and variability of practice. Res. Q. Exerc. Sport 61, 169–177. 10.1080/02701367.1990.10608671 2094928

[B74] Shmuelof L. Krakauer J. W. Mazzoni P. (2012). How is a motor skill learned? Change and invariance at the levels of task success and trajectory control. J. Neurophysiol. 108, 578–594. 10.1152/jn.00856.2011 22514286 PMC3404800

[B75] Sing G. C. Smith M. A. (2010). Reduction in learning rates associated with anterograde interference results from interactions between different timescales in motor adaptation. PLoS Comput. Biol. 6:e1000893. 10.1371/journal.pcbi.1000893 20808880 PMC2924244

[B76] Smith M. A. Ghazizadeh A. Shadmehr R. (2006). Interacting adaptive processes with different timescales underlie short-term motor learning. PLoS Biol. 4:e179. 10.1371/journal.pbio.0040179 16700627 PMC1463025

[B77] Spong M. W. Hutchinson S. Vidyasagar M. (2006). Robot Modeling and Control. Hoboken, NJ: John Wiley & Sons.

[B78] Sutskever I. Martens J. Dahl G. Hinton G. (2013). On the importance of initialization and momentum in deep learning, in International Conference on Machine Learning.

[B79] Takiyama K. Hirashima M. Nozaki D. (2015). Prospective errors determine motor learning. Nat. Commun. 6:5925. 10.1038/ncomms6925 25635628 PMC4316743

[B80] Thoroughman K. A. Shadmehr R. (2000). Learning of action through adaptive combination of motor primitives. Nature 407, 742–747. 10.1038/35037588 11048720 PMC2556237

[B81] Todorov E. (2004). Optimality principles in sensorimotor control. Nat. Neurosci. 7, 907–915. 10.1038/nn1309 15332089 PMC1488877

[B82] Turnham E. J. Braun D. A. Wolpert D. M. (2012). Facilitation of learning induced by both random and gradual visuomotor task variation. J. Neurophysiol. 107, 1111–1122. 10.1152/jn.00635.2011 22131385 PMC3289458

[B83] van Beers R. J. Baraduc P. Wolpert D. M. (2002). Role of uncertainty in sensorimotor control. Philos. Trans. R. Soc. Lond. B. Biol. Sci. 357, 1137–1145. 10.1098/rstb.2002.1101 12217180 PMC1693018

[B84] Wainscott S. K. Donchin O. Shadmehr R. (2005). Internal models and contextual cues: encoding serial order and direction of movement. J. Neurophysiol. 93, 786–800. 10.1152/jn.00240.2004 15385598

[B85] Wang J. Sainburg R. L. (2007). The dominant and nondominant arms are specialized for stabilizing different features of task performance. Exp. Brain Res. 178, 565–570. 10.1007/s00221-007-0936-x 17380323 PMC10702172

[B86] Wei K. Kording K. (2009). Relevance of error: what drives motor adaptation? J. Neurophysiol. 101, 655–664. 10.1152/jn.90545.2008 19019979 PMC2657056

[B87] Witney A. G. Wolpert D. M. (2003). Spatial representation of predictive motor learning. J. Neurophysiol. 89, 1837–1843. 10.1152/jn.00929.2002 12686568

[B88] Wolpert D. M. Kawato M. (1998). Multiple paired forward and inverse models for motor control. Neural Netw. 11, 1317–1329. 10.1016/S0893-6080(98)00066-5 12662752

[B89] Wu H. G. Miyamoto Y. R. Gonzalez Castro L. N. Olveczky B. P. Smith M. A. (2014). Temporal structure of motor variability is dynamically regulated and predicts motor learning ability. Nat. Neurosci. 17, 312–321. 10.1038/nn.3616 24413700 PMC4442489

[B90] Wulf G. Schmidt R. A. (1997). Variability of practice and implicit motor learning. J. Exp. Psychol. Learn. Memory Cogn. 23:987. 10.1037/0278-7393.23.4.987

[B91] Yadav V. Sainburg R. L. (2014). Limb dominance results from asymmetries in predictive and impedance control mechanisms. PLoS ONE 9:e93892. 10.1371/journal.pone.0093892 24695543 PMC3973649

