# Supplementary figures and images for: Direction-Specific Iterative Tuning of Motor Commands With Local Generalization During Randomized Reaching Practice Across Movement Directions
Source: Front Neurorobot. 2021 Oct 29;15:651214. doi: 10.3389/fnbot.2021.651214 (PMC8586720; doi:10.3389/fnbot.2021.651214)

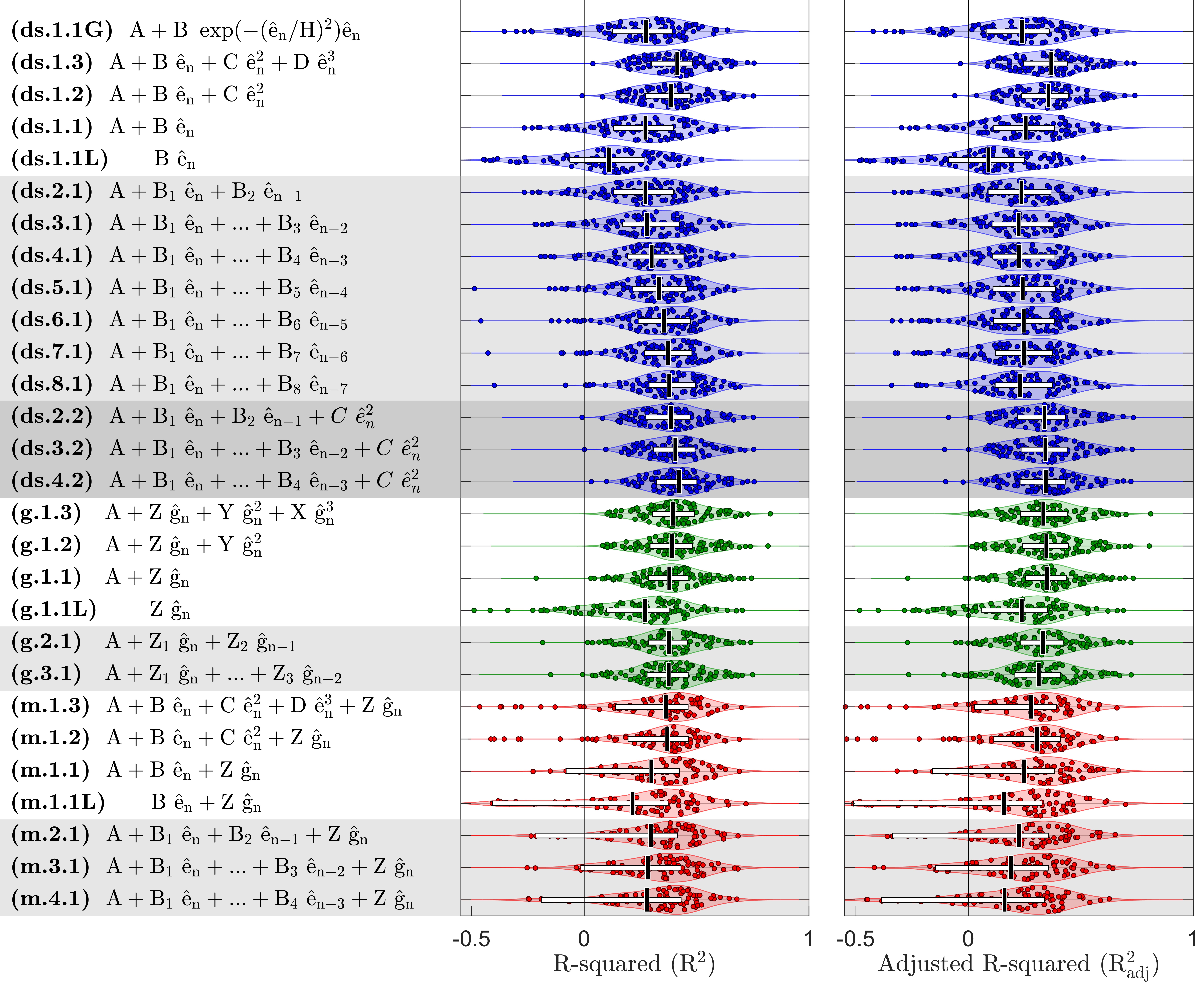

Supplement: Supplementary Figure 1 — Summary of regression statistics using the coefficient of determination (R2 and Radj2). The model structures listed on the left represent the inter-trial change in movement error in response to the visually perceived error during the initial launch of movement. Each colored circle represents its respective statistics for a particular learning task for subjects (102 estimates from 8 learning tasks x 15 subjects minus learning tasks with EA-gain = 0). Shaded colored regions show kernel density estimates for the distribution. Horizontal white bars and vertical black bars represent inter-quartile range and median across all subjects and learning tasks (blue = direction-specific model; green = generalizing model; red = mixed model). [file Image_1.jpg]

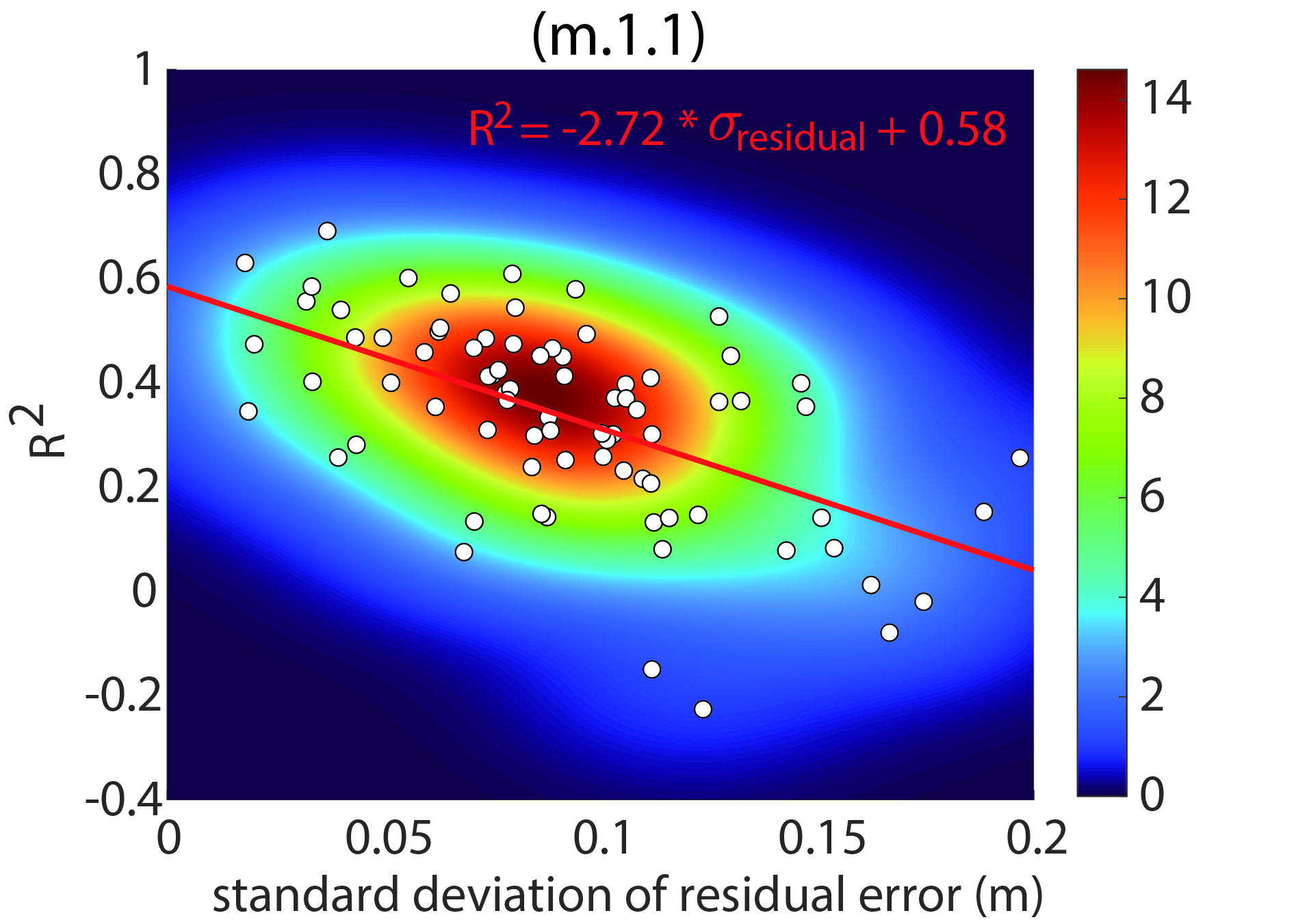

Supplement: Supplementary Figure 2 — Relationship between R2 and standard deviation of residual error for the first-order mixed model (m.1.1). White circles represent learning tasks for subjects. Shaded colored region represents the kernel density estimate of the data. Red curve represents the linear regression between R2 and standard deviation of residual error (F-test on the linear regression model against constant model yielded p = 2.64*10−8). Low SNR (approximated as high standard deviation of residual error) deteriorated the fit quality (R2) for the first-order mixed model (m.1.1). [file Image_2.jpg]

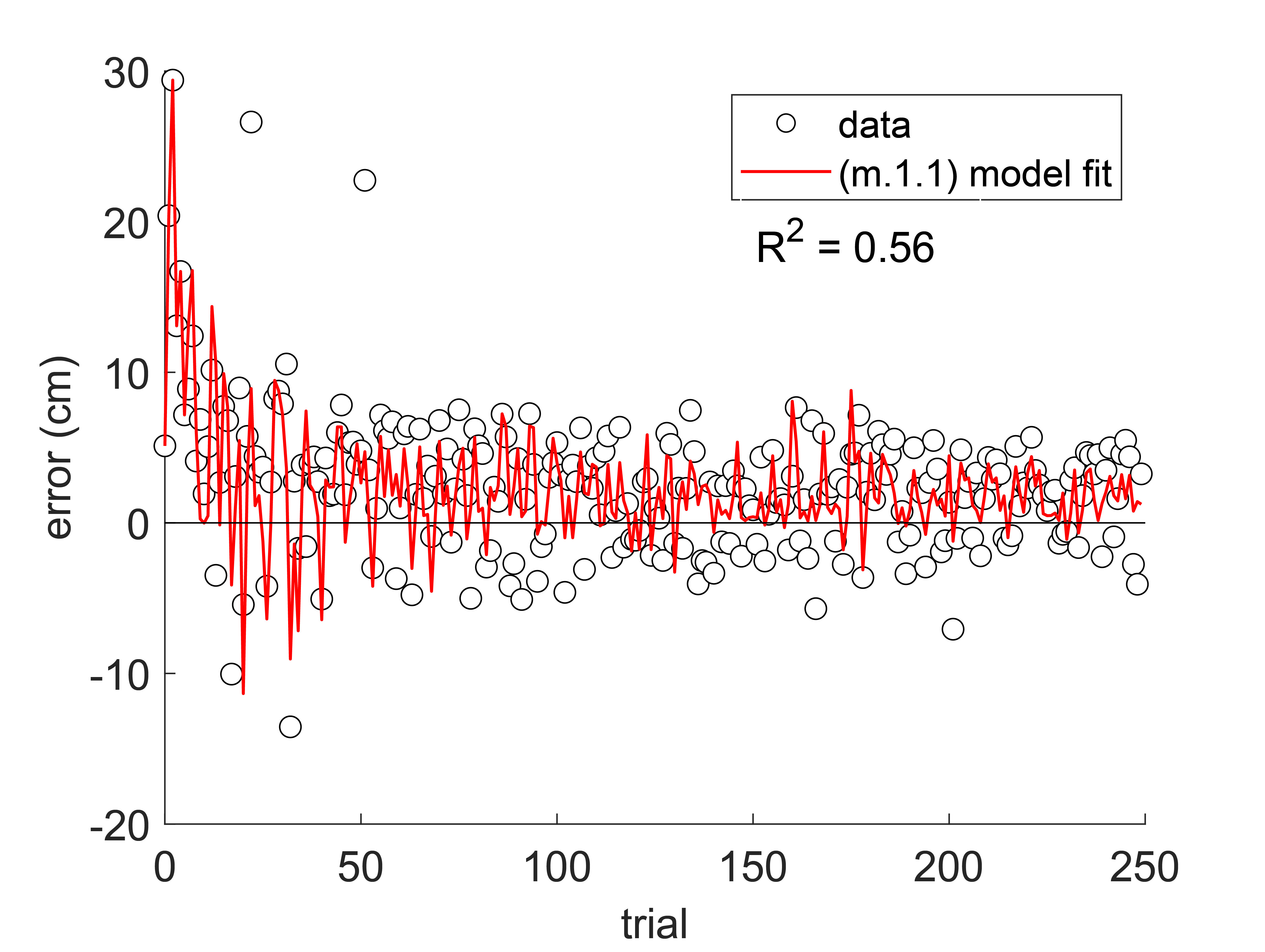

Supplement: Supplementary Figure 5 — An example of the best model fit to learning data from a subject. Circles are the errors performed by a subject during the learning task 4 with EA{gain 1, offset 0}, and red curve is the fit of the affine mixed model (m.1.1). This richness of behavior, such as the oscillation about zero, is possible due to the multiple factors, a signed error metric, and iterative incremental learning considered in the model. [file Image_5.jpg]

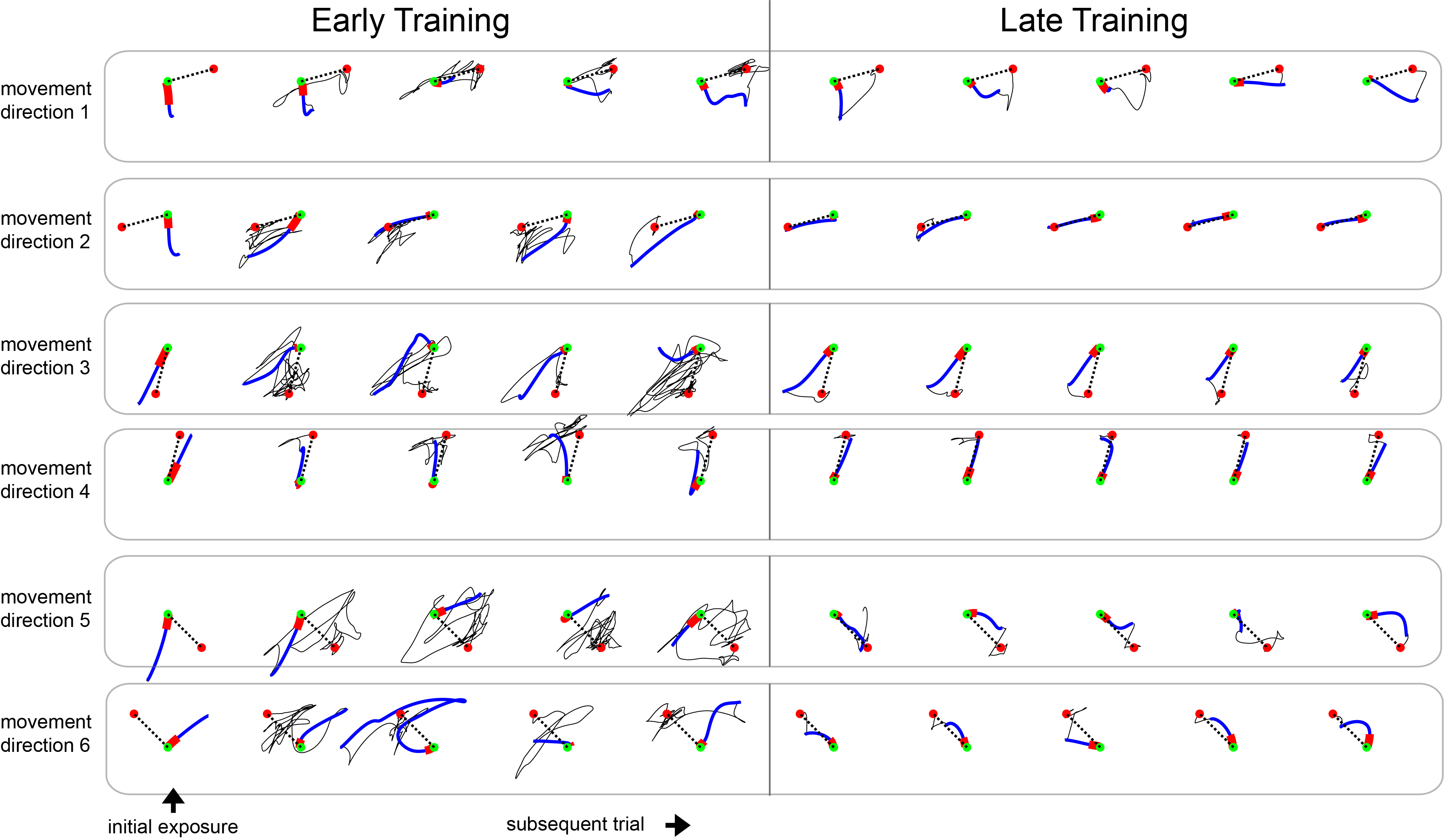

Supplement: Supplementary Figure 6 — Representative examples of movement paths from a subject practicing the learning task 3 with EA{gain 1,offset 1}. Green and red circles represent the start and target positions for the movements (15 cm apart). The dotted black line is the ideal straight line. Blue segment is the initial launch of the movement, and red segment is the first 150 ms of the initial launch. Solid Black line is the feedback correction phase of the movement where the subject navigated to the target after the initial launch. Note that the feedback correction phase is highly random during the early training and is greatly reduced and systematic during the late training. [file Image_6.jpg]
